# Supplementary material for: Are free school meals failing families? Exploring the relationship between child food insecurity, child mental health and free school meal status during COVID-19: national cross-sectional surveys
Source: BMJ Open. 2022 Jun 8;12(6):e059047. doi: 10.1136/bmjopen-2021-059047 (PMC9184996; doi:10.1136/bmjopen-2021-059047)
Supplement: Supplementary data [file bmjopen-2021-059047supp001.pdf]

Supplemental file 1. Characteristics of the survey population by participants responding to both surveys or to the first survey period (August-September 2020).

	Total sample n=2166		August-September 2020 only N=858		Responded to both surveys N=206		
	N	Mean (SD)/%	N	Mean (SD)/%	N	Mean (SD)/%	p-value
**Parent responses**							
**Parent age**							**0.07**
18-24	8	0.4	1	0.1	0	0	
25-34	268	12.4	112	13.1	18	8.7	
35-44	923	42.6	377	43.9	78	37.9	
45-54	762	35.2	292	34	88	42.7	
55-64	205	9.5	76	8.9	22	10.7	
Missing	-	-	-	-	-	-	
**Parent occupation**							**0.4**
Higher	1341	61.9	556	65	126	61	
Lower	825	38.1	302	35	80	39	
Missing	-	-	-	-	-	-	
**Geographical region**							**0.05**
East Midlands	158	7.3	68	7.9	6	2.9	
Eastern	196	9	72	8.4	20	9.7	
London	282	13	117	13.6	20	9.7	
North East	92	4.2	38	4.4	7	3.4	
North West	240	11.1	95	11.1	25	12.1	
Northern Ireland	73	3.4	27	3.1	9	4.1	
Scotland	161	7.4	59	6.9	25	12.1	
South East	300	13.9	122	14.2	26	12.6	
South West	197	9.1	76	8.9	15	7.3	
Wales	109	5	43	5.0	13	6.3	
West Midlands	182	8.4	57	7.8	24	11.7	
Yorkshire & Humberside	176	8.1	74	8.6	16	7.8	
Missing	-	-	-	-	-	-	
**Number in household**							**0.4**
2	160	7.4	66	7.7	8	3.9	
3	624	28.8	248	28.9	62	30.1	
4	939	43.4	361	42.1	89	43.2	
5	318	14.7	124	14.5	33	16	
6+	125	5.8	59	6.9	14	6.8	
Missing	-	-	-	-	-	-	
**Child ethnicity**							**0.6**
Asian	245	11.4	95	11.1	22	10.8	
Other†	209	9.7	69	8.1	21	10.3	
White	1691	78.8	687	80.8	161	78.9	
Missing	21	-	8	-	2	-	

**Child age (years)**	2166	12.4 (3.2)	858	12.4 (3.2)	206	12.5 (3.2)	**0.7**
Missing	-	-	-	-	-	-	
**Child sex**							**0.4**
Female	1076	49.7	422	49	109	53	
Male	1090	50.3	436	51	97	47	
Missing	-	-	-	-	-	-	
**Child receives FSM**							**0.08**
Yes	675	31.5	260	31	50	24	
No	1467	68.5	587	79	156	76	
Missing	24	-	11	-	-	-	
**Child responses**							
**Potential food insecurity**							**0.2**
Yes	431	20.6	165	20	31	15	
No	1659	79.4	667	80	171	85	
Missing	76	-	26	-	4	-	
**Any food bank use**							**0.002**
Yes	561	25.9	224	26	32	16	
No	1605	74.1	634	74	174	84	
Missing	-	-	-	-	-	-	
**Food insecure***							**0.01**
Yes	763	35.2	298	35	52	25	
No	1403	64.8	560	65	154	75	
Missing	-	-	-	-			
**Find FSM embarrassing**							**0.8**
Yes	62	9.7	26	11	4	8.2	
No	578	90.3	214	89	45	91.8	
Missing	1526	-	618	-	157	-	

†The Other ethnicity category includes the following groups: Black African, Black Caribbean, other Black background, mixed, and other background.

*Defined as responding affirmatively to any of the 6 potential food insecurity questions or indicated any food bank use
