# Supplementary material for: Are free school meals failing families? Exploring the relationship between child food insecurity, child mental health and free school meal status during COVID-19: national cross-sectional surveys
Source: BMJ Open. 2022 Jun 8;12(6):e059047. doi: 10.1136/bmjopen-2021-059047 (PMC9184996; doi:10.1136/bmjopen-2021-059047)
Supplement: Supplementary data [file bmjopen-2021-059047supp002.pdf]

Supplemental file 2. Characteristics of the survey population by survey period

	Total sample n=2166		August-September 2020 N=858		January-February 2021 N=1308		
	N	Mean (SD)/%	N	Mean (SD)/%	N	Mean (SD)/%	p-value
**Parent responses**							
**Parent age**							**0.3**
18-24	8	0.4	1	0.1	7	0.5	
25-34	268	12.4	112	13.1	156	11.9	
35-44	923	42.6	377	43.9	546	41.7	
45-54	762	35.2	292	34	470	35.9	
55-64	205	9.5	76	8.9	129	9.9	
Missing	-	-	-	-	-	-	
**Parent occupation**							**0.03**
Higher	1341	61.9	556	65	785	60	
Lower	825	38.1	302	35	423	40	
Missing	-	-	-	-	-	-	
**Geographical region**							**1**
East Midlands	158	7.3	68	7.9	90	6.9	
Eastern	196	9	72	8.4	124	9.5	
London	282	13	117	13.6	165	12.6	
North East	92	4.2	38	4.4	54	4.1	
North West	240	11.1	95	11.1	145	11.1	
Northern Ireland	73	3.4	27	3.1	46	3.5	
Scotland	161	7.4	59	6.9	102	7.8	
South East	300	13.9	122	14.2	178	13.6	
South West	197	9.1	76	8.9	121	9.3	
Wales	109	5	43	5.0	66	5.0	
West Midlands	182	8.4	57	7.8	115	8.8	
Yorkshire & Humberside	176	8.1	74	8.6	102	7.8	
Missing	-	-	-	-	-	-	
**Number in household**							**0.4**
2	160	7.4	66	7.7	94	7.2	
3	624	28.8	248	28.9	376	28.7	
4	939	43.4	361	42.1	578	44.2	
5	318	14.7	124	14.5	194	14.8	
6+	125	5.8	59	6.9	66	5.0	
Missing	-	-	-	-	-	-	
**Child ethnicity**							**0.1**
Asian	245	11.4	95	11.1	151	12	
Other†	209	9.7	69	8.1	140	11	
White	1691	78.8	687	80.8	1004	78	
Missing	21	-	8	-	13	-	
**Child age (years)**	2166	12.4 (3.2)	858	12.4 (3.2)	1308	12.4 (3.2)	

Missing	-	-	-	-	-	-	
**Child sex**							**0.7**
Female	1076	49.7	422	49	654	50	
Male	1090	50.3	436	51	654	50	
Missing	-	-	-	-	-	-	
**Child receives FSM**							**0.5**
Yes	675	31.5	260	31	415	32	
No	1467	68.5	587	79	880	68	
Missing	24	-	11	-	13	-	
**Child responses**							
**Potential food insecurity**							**0.5**
Yes	431	20.6	165	20	266	21	
No	1659	79.4	667	80	992	79	
Missing	76	-	26	-	60	-	
**Any food bank use**							**0.9**
Yes	561	25.9	224	26	337	26	
No	1605	74.1	634	74	971	74	
Missing	-	-	-	-	-	-	
**Food insecure***							**0.7**
Yes	763	35.2	298	35	465	36	
No	1403	64.8	560	65	843	64	
Missing	-	-	-	-	-	-	
**Find FSM embarrassing**							**0.3**
Yes	62	9.7	26	11	36	9.0	
No	578	90.3	214	89	364	91	
Missing	1526	-	618	-	908	-	
**Stressed/worried**[ǂ]							-
Every/most days	236	18	-	-	236	18	
Some/rarely	1053	82	-	-	1053	82	
Missing	19	-	-	-	19	-	

†The Other ethnicity category includes the following groups: Black African, Black Caribbean, other Black background, mixed, and other background.

*Defined as responding affirmatively to any of the 6 potential food insecurity questions or indicated any food bank use

ǂResponses available only among a children participating in the January-February 2021 survey.

p-value differences between survey periods
