# Supplementary figures and images for: Are free school meals failing families? Exploring the relationship between child food insecurity, child mental health and free school meal status during COVID-19: national cross-sectional surveys
Source: BMJ Open. 2022 Jun 8;12(6):e059047. doi: 10.1136/bmjopen-2021-059047 (PMC9184996; doi:10.1136/bmjopen-2021-059047)

**Supplementary file 3.** DAG for food insecurity and child mental health.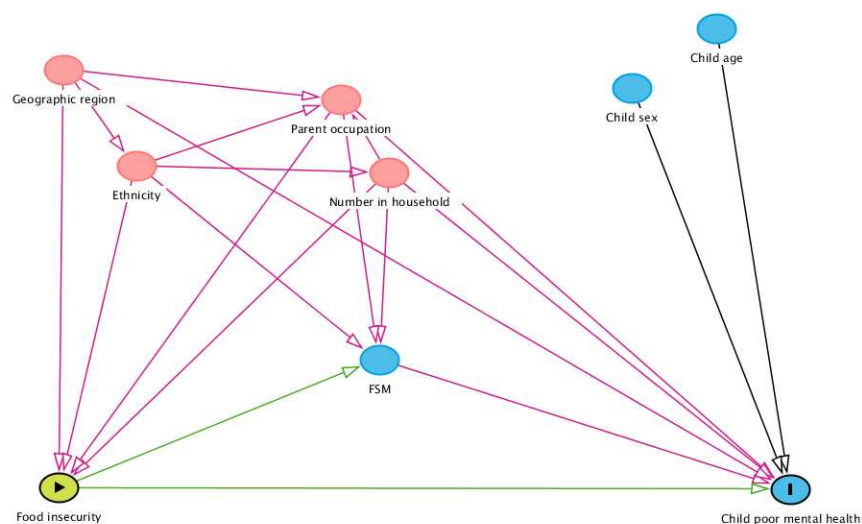

Supplement: Supplementary data [file bmjopen-2021-059047supp003.pdf]
