# Supplementary material for: Are free school meals failing families? Exploring the relationship between child food insecurity, child mental health and free school meal status during COVID-19: national cross-sectional surveys
Source: BMJ Open. 2022 Jun 8;12(6):e059047. doi: 10.1136/bmjopen-2021-059047 (PMC9184996; doi:10.1136/bmjopen-2021-059047)
Supplement: Author's manuscript [file bmjopen-2021-059047.draft_revisions.pdf]

BMJ Open is committed to open peer review. As part of this commitment we make the peer review history of every article we publish publicly available.

When an article is published we post the peer reviewers' comments and the authors' responses online. We also post the versions of the paper that were used during peer review. These are the versions that the peer review comments apply to.

The versions of the paper that follow are the versions that were submitted during the peer review process. They are not the versions of record or the final published versions. They should not be cited or distributed as the published version of this manuscript.

BMJ Open is an open access journal and the full, final, typeset and author-corrected version of record of the manuscript is available on our site with no access controls, subscription charges or pay-per-view fees (http://bmjopen.bmj.com).

If you have any questions on BMJ Open's open peer review process please email
info.bmjopen@bmj.com

BMJ Open

Are Free School Meals failing families? Exploring the relationship between child food insecurity, child mental health and Free School Meal status during COVID-19: national cross-sectional surveys

Journal:	*BMJ Open*
Manuscript ID	bmjopen-2021-059047
Article Type:	Original research
Date Submitted by the Author:	05-Nov-2021

Complete List of Authors:	Yang, Tiffany; Bradford Teaching Hospitals NHS Foundation Trust<br>Power, Madeleine; University of York<br>Moss, Rachael ; Bradford Teaching Hospitals NHS Foundation Trust<br>Burton, Wendy; University of York<br>Lockyer, Bridget; Bradford Institute for Health Research<br>Doherty, Bob; University of York, The York Management School<br>Bryant, Maria; University of York, Health Sciences; Hull York Medical School

Keywords:	MENTAL HEALTH, COVID-19, PUBLIC HEALTH

SCHOLARONE™
Manuscripts

I, the Submitting Author has the right to grant and does grant on behalf of all authors of the Work (as defined in the below author licence), an exclusive licence and/or a non-exclusive licence for contributions from authors who are: i) UK Crown employees; ii) where BMJ has agreed a CC-BY licence shall apply, and/or iii) in accordance with the terms applicable for US Federal Government officers or employees acting as part of their official duties; on a worldwide, perpetual, irrevocable, royalty-free basis to BMJ Publishing Group Ltd ("BMJ") its licensees and where the relevant Journal is co-owned by BMJ to the co-owners of the Journal, to publish the Work in this journal and any other BMJ products and to exploit all rights, as set out in our licence.

The Submitting Author accepts and understands that any supply made under these terms is made by BMJ to the Submitting Author unless you are acting as an employee on behalf of your employer or a postgraduate student of an affiliated institution which is paying any applicable article publishing charge ("APC") for Open Access articles. Where the Submitting Author wishes to make the Work available on an Open Access basis (and intends to pay the relevant APC), the terms of reuse of such Open Access shall be governed by a Creative Commons licence – details of these licences and which Creative Commons licence will apply to this Work are set out in our licence referred to above.

Other than as permitted in any relevant BMJ Author's Self Archiving Policies, I confirm this Work has not been accepted for publication elsewhere, is not being considered for publication elsewhere and does not duplicate material already published. I confirm all authors consent to publication of this Work and authorise the granting of this licence.

For peer review only

Are Free School Meals failing families? Exploring the relationship between child food insecurity, child mental health and Free School Meal status during COVID-19: national cross-sectional surveys

Tiffany C Yang[1] (0000-0003-4549-7850) Principal research fellow, Madeleine Power[2] (0000-0002-9571-1782) Research fellow, Rachael H Moss[1] (0000-0001-6160-8536) Research fellow, Bridget Lockyer[1] (0000-0002-2195-5549) Senior research fellow, Wendy Burton[2] (0000-0001-7885-5971) Research associate, Bob Doherty[3] (0000-0001-6724-7065) Professor, Maria Bryant[2,4] (0000-0001-7690-4098) Reader
[1]Bradford Institute of Health Research, Bradford Teaching Hospital NHS Foundation Trust, Bradford, BD9 6RJ, UK
[2]Department of Health Sciences, University of York, York, YO10 5DD, UK
[3]University of York Management School, University of York, York, YO10 5DD, UK
[4]Department of Health Sciences and the Hull York Medical School, University of York, York, YO10 5DD, UK

Correspondence to:
Tiffany Yang
Bradford Institute for Health Research
Bradford Royal Infirmary
Duckworth Lane
Bradford BD9 6RJ
Tiffany.yang@bthft.nhs.uk

Abstract

Objective: Food insecurity is linked to poor health and wellbeing in children, and rising prevalence rates have been exacerbated by COVID-19. Free School Meals (FSM) are considered a critical tool for reducing the adverse effects of poverty but apply a highly restrictive eligibility criteria. This study examined levels of food security and FSM status to support decision making regarding increasing the current eligibility criteria.

Design: Two cross-sectional national surveys administered in August-September 2020 and January-February 2021 were used to examine the impact of COVID-19 on the food experiences of children and young people.

Setting: United Kingdom.

Participants: 2,166 children (aged 7-17 years) and parents/guardians.

Main outcome measures: Participant characteristics were described by food security and FSM status; odds of poor mental health by food security and FSM status, adjusted for confounding variables.

Results: We observed food insecurity among both children who were or were not in receipt of FSM: 23% of children not receiving FSM were food insecure. Children who were food insecure had worse mental health compared to children who were food secure (Odds Ratio [OR]: 5.24, 95% Confidence Interval [95% CI]: 3.84 to 7.20); and this was lower among children receiving FSM (OR: 1.99, 95% CI: 1.42 to 2.78) compared to those who were not.

Conclusion: Many children experienced food insecurity regardless of whether they received FSM suggesting the eligibility criteria needs to be widened to prevent overlooking those in need.

For peer review only - http://bmjopen.bmj.com/site/about/guidelines.xhtml

Keywords

Food insecurity, child food insecurity, free school meals, mental health, COVID-19.

Strengths and limitations

- Geographic and demographically-representative sample with wide range of ages of children
- Child-reported measures of food insecurity and mental health
- Parents may have helped their child complete the survey and influenced responses among younger children
- Analyses were not stratified by age though food insecurity may be experienced differently between those of younger and older ages
- We were unable to differentiate between eligibility and uptake

For peer review only

Introduction

The past decade has witnessed sharp rises in the use of food banks by households with children, suggesting that child food insecurity is rising[1]. Food insecurity can broadly be defined as uncertainty around the quality and quantity of food available[2]. Data from the Family Resource Survey (FRS) indicates that households with children are at particular risk of food insecurity in the UK [2–4]. In 2019/2020, five million people in the UK (8%) were in food insecure households, of whom 13% were children, 8% were working age adults, and 2% were pensioners [3]. Food insecurity has considerable implications for children, including associations with lower vegetable intake, higher added sugar intake [5,6], increased risk of obesity [4,7–9] and poorer academic performance [10]. There is a growing body of literature - almost entirely from North America - evidencing an association between the experience of food insecurity and an increase in the risk of mental health issues for children and adolescents [11–16]. Children and teenagers experiencing food insecurity report lower life satisfaction [12], and have a higher probability of seeing a psychologist and finding it difficult to make friends [17]. Evidence suggests that rates of depression [18], stress and anxiety are higher for children living in food insecure households [13,19,20]. Households with children have been particularly badly affected by the social and economic fallout of the COVID-19 pandemic. In the first six months of the pandemic, 12% of adults living with children reported skipping meals because they could not afford or access food, while 4% of adults with children reported going for a whole day without eating [21]. Food banks also reported a sharp rise in access by households with young children. Between early and mid-2020, The Trussell Trust food bank network supported 370,000 households, of which 320,000 were families with children. The proportion of couples with children referred to a food bank increased from 19% in early 2020 to 24% during the COVID-19 pandemic in mid-2020[22].

Free school meals (FSM) are considered to be a critical tool for mitigating the negative health effects of child poverty among low-income families. Children receiving FSM obtain a higher proportion of their daily energy and nutrient intakes from their school meals compared to those who pay [23,24] and FSM may therefore improve health and wellbeing and reduce health inequalities [25,26]. In England, FSM are currently a statutory entitlement available to eligible pupils, which include all infant school children (reception, year 1, and year 2) in state-funded schools (as part of the Education Act, 1944) [27]; and pupils in year 3 and upwards (junior school and secondary school pupils) whose parents meet income-defined eligibility criteria[1] and claim for FSM. As of 1 October 2020, there were 1.63 million pupils known to be eligible for FSM [28], an increase in the proportion eligible to 19.7% of all state-funded pupils from 17.3% in January 2020 and 15.4% in January 2019. This increase is likely due to increased unemployment during the COVID-19 pandemic rendering more children eligible for FSM, alongside increased uptake due to greater media attention and awareness of FSM.

FSM receipt can be considered a marker of poverty due to its restrictive eligibility criteria and children who receive FSM are likely to be living in low income households. The COVID-19 pandemic has exposed and amplified pre-existing concerns about the restrictive eligibility criteria for FSM (for pupils above year 2) and low uptake of FSM among eligible families (both before and after

[1] Parents currently meet the eligibility criteria if they receive: Income Support; Income-based Jobseekers Allowance; Income-related Employment and Support Allowance; Support under Part VI of the Immigration and Asylum Act 1999; the guaranteed element of State Pension Credit; Child Tax Credit (provided they are not also entitled to Working Tax Credit and have an annual gross income of no more than £16,190); Working Tax Credit run-on (paid for four weeks after a person stops qualifying for Working Tax Credit); and Universal Credit (with household income of less than £7,400 a year after tax and not including any benefits).

For peer review only - http://bmjopen.bmj.com/site/about/guidelines.xhtml

registration)[29]. The effects of the pandemic have been highly unequal, according to income, ethnicity, gender and health status [30–36]. There is evidence to suggest that low income families have been particularly negatively affected by the social and economic circumstances of the pandemic [37–39] and yet have thus far been largely neglected in the Governmental policy response. Emerging evidence suggests that families just outside of the eligibility criteria for FSM have struggled to afford food during the pandemic, potentially exacerbating child food insecurity [40]. However, this has not yet been formally assessed. This paper addresses this important and urgent research gap, examining the relationship between child food insecurity and families in receipt of and not in receipt of FSM during the pandemic. Given the known negative effects of food insecurity on child mental health and educational outcomes [10,11,41], the paper also looks at child mental health in the context of child food insecurity and receipt of FSM.

Methods

Study population and survey design

Data were taken from two Food Foundation commissioned surveys (ChildWise) conducted in the course of the COVID-19 pandemic to examine its impact on children and young people's COVID-19 food experiences. The first (August-September 2020) and second surveys (January-February 2021) were carried out online using a carefully constructed framework to ensure a geographic and demographic representative sample of adults living in the UK with children and young people aged 7-17 years.

The online panel used by ChildWise is a member of the ESOMAR (European Society of Opinion and Marketing Research) organisation and endeavours to be as representative as possible. This panel is the largest in the UK and globally. To achieve representative quotas, the panel's profiling data was first used to target the more difficult-to-reach demographics before targeting other groups. Samples were recruited to be representative by region, broad ethnic group, and spread evenly by age and gender.

Surveys were completed by parents or guardians (hereafter "parents") of children with a section to be completed by children with the aid of parents if required. Parents were asked to list the ages and genders of all children in the household and one child was initially randomly allocated to complete the child portion of the survey. Parents completed questions on socio-demographic details and were asked to complete information about up to two of their children's FSM status, age, and gender. Towards the end of the fieldwork period, children were non-randomly assigned to complete the child portion of the survey based on fulfilling any remaining quotas of age, gender, and geographic region. The children's section included questions on perception of FSM, food insecurity and food bank use. In the second survey, additional questions on the child's mental health were included in the children's section.

Sociodemographic characteristics

We collapsed ethnicity from 12 categories into three: White (White British; Other White Background); Asian (Bangladeshi; Chinese; Indian; Pakistani; Other Asian Background) and Other (Black African; Black Caribbean; Other Black Background; Mixed background). Participants who chose "Prefer not to answer" were coded to missing.

Parental occupation was collapsed from 12 categories into two: Higher (Supervisory or clerical/ junior managerial/ professional/ administrative; Intermediate managerial/ professional/ administrative; Higher managerial/ professional/ administrative; Student) and Lower (Semi or unskilled manual work; Skilled manual worker; Casual worker - not in permanent employment; Housewife/Homemaker; Retired and living on state pension; Unemployed or not working due to long-term sickness; Full-time carer of other household member; Other).

For peer review only - http://bmjopen.bmj.com/site/about/guidelines.xhtml

Free School Meals

Parents were asked whether their child was currently registered for FSM. Responses were coded to "Yes" if parents responded "Yes" and "No" if parents responded "No". Responses of "Don't know" and "Prefer not to say" were coded to missing. Responses were similar to the question asked of children ("Thinking about when you have lunch at school, do you have Free School Meals?").

Food insecurity

Participants were asked to think about being at home during the summer holidays (August-September 2020 survey) or the Christmas holidays and recent lockdown (January-February 2021 survey) and asked to respond to several statements regarding potential food insecurity. Participants were categorised as having "potential food insecurity" if they responded "Yes" to any of the following 6 statements: (1) Sometimes I was hungry but didn't eat because I didn't want to use up the food we had; (2) Sometimes I was hungry but didn't eat because we didn't have enough food in the house; (3) Sometimes my parents didn't eat because we didn't have enough food in the house; (4) Sometimes we had to eat less and make food last longer because we didn't have the money to buy more; (5) Sometimes we didn't eat proper meals because we didn't have enough money to buy more food; (6) Sometimes I ate at friend's houses because we didn't have enough food in the house. Participants were categorised as not having potential food insecurity if they responded "Yes" to "I always had enough food to eat". There were no participants who responded affirmatively to both "I always had enough to eat" and any of the other 6 statements. Participants who responded affirmatively to "Don't know" or "Prefer not to say" were coded to missing.

Participants were also asked to respond to several statements regarding food bank use. They were coded to any food bank use if they responded "Yes" to having visited a food bank by themselves or if their family visited a food bank or coded to "No" if they responded "No, we didn't visit a food bank". Remaining participants were coded as not having used a food bank.

A dichotomous variable of "food insecurity" was then generated and included participants who were identified as having "potential food insecurity" (from the 6 questions) or indicated any food bank use. Participants who did not have "potential food insecurity" and did not indicate any food bank use were considered to be "food secure".

Mental health

Children were asked how often they felt stressed or worried in the past month and were categorised as being stressed or worried "Every/most days" if they said they were worried "every day" or "most days". Children were categorised as being stressed or worried "Some/rarely" if they said they were worried "Some days", "Rarely", or "I have not felt stressed once in the last month".

Data analysis

All analyses were conducted in R version 4.0.2[42]. Responses were combined and analysed across both surveys. A small number of participants responded to both surveys (n=206). Their responses were removed from the first survey so that they were present in the analysis only once. Main analyses were conducted on a sample size of n=2,166.

Participant responses were described using mean (standard deviation [SD]) for continuous measures and number (n) and percentage (%) for categorical measures. We constructed 4 groups: (1) children with food insecurity who had FSM; (2) children with food insecurity who did not have FSM; (3) children without food insecurity who had FSM; (4) children without food insecurity who did not have FSM.

Differences between participant characteristics and responses to food insecurity questions, food bank use, and derived food insecurity by children who received or did not receive FSM were assessed using χ^2 or Fisher's exact tests. A significant p-value ($p<0.05$) indicates that there is a difference between the characteristics by food security status among children who receive FSM and

For peer review only - http://bmjopen.bmj.com/site/about/guidelines.xhtml

among children who do not receive FSM. In the subset of survey questions on children's mental health, unadjusted and adjusted logistic regression was used to report odds of the child reporting feeling stressed or worried "every/most" days compared to "some/rarely" days regressed on food insecurity and FSM. A directed acyclic diagram (DAG) was drawn to assist in the selection of covariates (Supplementary file 1); confounding variables included in adjusted analyses of food insecurity with child mental health were ethnicity, parent occupation, and household occupancy. Variables included in adjusted analyses of FSM with child mental health were ethnicity, food insecurity, parent occupation, and household occupancy. Child age and sex were additionally adjusted to improve precision. Adjusted regressions can be interpreted as odds of children feeling stressed "every/most" days of the week for food insecure compared to food secure children or for children who received FSM compared to children who did not receive FSM, adjusting for covariates.

Results

Participant characteristics

The majority (78%) of parent respondents were aged 35-54 years old, were professionally employed (61.9%), and lived in households with two or three people (72%) (Table 1). The majority of children were White (78.8%) or Asian (11.4%) and female (57.2%) and just under a third of parents reported that their child received FSM (32%). Over a fifth of children reported any measure of food insecurity (21%) and over a quarter reported that they or their family had visited a food bank (25.9%), placing over a third of children living with food insecurity according to our definition (35%).

Among children who receive FSM, 60% were considered to have food insecurity (Table 2). Parents of children receiving FSM were younger, were more likely to be in a higher level of occupation, and less likely to be of White ethnicity. Over a fifth of children who did not receive FSM had food insecurity. Parents of children who did not receive FSM and had food insecurity were more likely to be younger than those not living with food insecurity. There was no difference in parental occupation between children who did not receive FSM and who lived with or without food insecurity with parents in both groups more likely to have a higher level of occupation.

Free school meals and potential food insecurity

More children receiving FSM reported any potential food insecurity measure than those not receiving FSM (42.8% among those who received FSM, 9.8% among those who did not; $p<0.05$; data not shown). Figure 1 shows the percentage of children responding affirmatively to each of the 6 potential food insecurity questions by FSM status. Children receiving FSM were more likely to respond affirmatively to these questions, though many who did not receive FSM also indicated potential food insecurity. Among all children, the most commonly chosen item reported was having to eat less in order to make food last longer due to a lack of money to buy more food.

Free school meals and food bank use

Both children who did and did not receive FSM reported visiting a food bank, whether by themselves or their family (Figure 2). Children who received FSM were more likely to have reported visiting a food bank by themselves or their family than children not receiving FSM. We found 2.7% of children not receiving FSM visited a food bank by themselves while 9.8% reported their families had visited a foodbank.

Child's mental health

For peer review only - http://bmjopen.bmj.com/site/about/guidelines.xhtml

Daily stress or worries were reported more frequently by children experiencing food insecurity compared to those food secure, whether in receipt of FSM (41%) or not (22%) (Figure 3). Children experiencing food insecurity had over five times the odds of feeling stressed every day or most days compared to children who were food secure (Table 3). This likelihood was reduced when taking food insecurity into account, with children who received FSM having twice the odds of feeling stressed or worried most days or every day compared to children who did not receive FSM.

Discussion

In this family-based survey measuring experiences of the COVID-19 pandemic, we found a substantial number of children experienced food insecurity (defined here as having ever visited a food bank or experienced any food insecurity measure) regardless of whether they were in receipt of FSM. Food insecurity and measures of potential food insecurity were highest among children in receipt of FSM, likely reflecting the very low income threshold for FSM (currently £7,400), meaning that outside of universal infant provision (in England and Scotland), it is largely children in the very poorest families who receive FSM. In a subset of children with mental health measures, we found that children who experienced food insecurity had increased odds of feeling stressed or worried on an almost-daily basis in the previous month compared to children who were food secure.

Children are often protected from hunger in families that experience food insecurity where parents report decreasing their own intake to shield their children [43–46]. We found that children reported their parents skipping meals due to a lack of food in the house. However, it was concerning that we also found children reporting hunger due to not having enough food in the house and almost a fifth of all children living with food insecurity reporting having to eat less to make food stretch. The high proportion of children with food insecurity reporting food bank use is consistent with other reports that have highlighted the impact of the pandemic on levels of food insecurity [1]. The consequences of the pandemic on financial stability and, therefore, food insecurity, have also impacted families who may not have been previously affected.

Studies have observed food insecurity among the employed and recent data in the UK Longitudinal Household Study on food insecurity during the pandemic suggests that, while risk of food insecurity increases more for those who were unemployed, those who are persistently employed were also at risk [47–50]. The pandemic has exposed the notion that food insecurity occurs primarily among the unemployed or those less-skilled professions; over 50% of children reporting food insecurity in our data had parents with higher/professional levels of occupation. Educational attainment and income are not necessarily protective of food insecurity. In the 2019/2020 FRS, it was reported that 8% of households where the head of household obtained A-levels or Scottish Highers and 4% of households with further education and university qualifications were food insecure [3]. Likewise, increasing income increased the food security but even among those with a gross weekly income of £1,000 or more only 96% had high food security (meaning 4% experienced food insecurity), compared to 74% of households with a total gross weekly income of less than £200 have high food security.

Over 25% of all children and almost 50% of children living with food insecurity reported their families visiting a food bank. Previous research has suggested that use of food banks by UK households experiencing food insecurity is low [51,52]; however, our study suggests that, in the context of the pandemic, food bank use may have become more common for families experiencing food poverty. Food banks are a short-term 'emergency' response and concerns have been raised about the nutritional quality [53,54]and cultural adequacy of the food provided [55]. The emergence and continuation of food banks and the growing number of food parcels they provide may be seen as an example of 'successful' self-organisation around a need and conveys a sense that something is being

For peer review only - http://bmjopen.bmj.com/site/about/guidelines.xhtml

done; however, it should be questioned whether it is the responsibility of the voluntary sector, rather than the Government, to provide access to something as basic as having food of sufficient quality and quantity. Food banks are often unable to provide fresh foods or ensure dietary requirements are met; continued reliance and widespread use of food banks, particularly among households with children, raises concerns about the long-term mental and physical implications for families relying on this form of 'emergency' support.

FSM are often seen as an essential tool for mitigating the effects of poverty experienced by children but provision is not universal or standardised across all nations, leading to unequal access (see Patrick et al., 2021). Our data reported more children registered for FSM (32%) compared to those reported eligible in autumn 2020 (19.7%) [28]. This may be reflected by an increase in newly-qualifying children for FSM as families lost income; in the first survey, over 40% of children registered for FSM had only recently started receiving FSM (i.e., were newly eligible that term) [21]. Once children age out of universal provision, stringent criteria must be met for children to receive FSM with many of the criteria being income-based, leading to only very low-income families being eligible and many low-income families going hungry. The eligibility is so restrictive that in our sample nearly half of families who are food insecure do not receive FSM; the eligibility threshold is set at an annual household income of less than £7,400 prior to benefits while parents receiving Working Tax Credit are ineligible for FSM support regardless of their level of income. However, as we have shown, a large proportion of children experiencing food insecurity as well as those in receipt of FSM have parents employed in professional-level occupations. This suggests that the financial circumstances of families of all income levels have been hard-hit by the pandemic and that the current criteria may not be suitable for assessing eligibility. In addition, our findings that children who were receiving FSM still reported hunger suggests that FSM provision may not be sufficient to ensure that children are adequately fed on a daily basis.

There is limited published research in the UK on the role food insecurity plays in children's mental health, and none on the role of FSM in mitigating the association between food insecurity and poor mental health among children. Emerging UK evidence suggests poorer wellbeing and increased emotional and behavioural problems among children who experience food insecurity. One UK study found 27% of 10-year old children experiencing food insecurity exhibited clinically significant behavioural problems compared to 10% of children who were food secure[56]. Our findings that children who experience food insecurity have worse mental health are therefore unsurprising, and in line with North American literature on food insecurity and child mental health[11–16]. This strength of association was reduced when we examined the relationship between receipt of FSM and children reporting feeling stressed or worried and accounting for food insecurity, suggesting that FSM helped alleviate the burden of poverty and food insecurity. However, children who received FSM still had higher odds of reporting feeling stressed or worried compared to children who did not receive FSM. This could potentially reflect the complex poverty-related stressors of living in a household eligible for FSM as well as the perceived stigma of receiving FSM[37,57]. Children who received FSM in this survey were asked whether they think it is embarrassing to have FSM and 11.5% of children living with food insecurity thought it was embarrassing compared to 4.1% of children who were food secure. This suggests children may carry an additional burden of stigma on top of inadequate food security.

There are multiple strengths and limitations to this study. This study used a geographic and demographically-representative sample with a wide range of ages and included measures of child-reported food insecurity combined with child reported mental health. However, we were unable to understand the degree to which parents may have helped their child complete the questions, and whether responses were given by them or were changed/given by their parents. This is more likely to have influenced responses from the younger children completing the survey. We also did not

For peer review only - http://bmjopen.bmj.com/site/about/guidelines.xhtml

stratify the sample to distinguish between children in primary or secondary school, which may reflect differences in how food insecurity is experienced, such as visiting a food bank themselves, as well as differential uptake in FSM regardless of eligibility. We were also unable to differentiate between eligibility and uptake and whether the 23% of children experiencing food insecurity but not receiving FSM were due to non-eligibility or from voluntary refusal as a result of stigma or other reasons for not participating when eligible, such as navigating the application process.

Our findings confirm a real need to reconsider the eligibility criteria currently set for the provision of FSM. A concerning number of children are experiencing food insecurity in families with higher/professional levels of education who are likely to be above the eligibility threshold for FSM. While more families can be helped by widening eligibility, and more work is needed to understand access and uptake of FSM, including potential barriers such as social shame, policies which provide universal coverage should be considered as the impact goes beyond food provision and eliminates the stigma that is associated with being eligible and receiving FSM.

For peer review only

References

1 Power M, Doherty B, Pybus K, *et al.* How Covid-19 has exposed inequalities in the UK food system: The case of UK food and poverty. *Emerald Open Res* 2020;**2**:11. doi:10.35241/emeraldopenres.13539.1
2 Bickel G, Nord M, Price C, *et al.* Guide to Measuring Household Food Security. 2000.
3 Department of Work and Pensions. Family Resources Survey: financial year 2019 to 2020. 2021. https://www.gov.uk/government/statistics/family-resources-survey-financial-year-2019-to-2020/family-resources-survey-financial-year-2019-to-2020
4 Taylor A, Loopstra R. Too Poor to Eat Food insecurity in the UK. *foodfoundation.org.uk* 2016;:1–12.www.foodfoundation.org.uk
5 Eicher-Miller HA, Zhao Y. Evidence for the age-specific relationship of food insecurity and key dietary outcomes among US children and adolescents. *Nutr Res Rev* 2018;**31**:98–113. doi:10.1017/S0954422417000245
6 Hanson KL, Connor LM. Food insecurity and dietary quality in US adults and children: a systematic review. *Am J Clin Nutr* 2014;**100**:684–92. doi:10.3945/ajcn.114.084525
7 Yang TC, Sahota P, Pickett KE, *et al.* Association of food security status with overweight and dietary intake: exploration of White British and Pakistani-origin families in the Born in Bradford cohort. *Nutr J* 2018;**17**:48. doi:10.1186/s12937-018-0349-7
8 Aceves-Martins M, Cruickshank M, Fraser C, *et al.* Child food insecurity in the UK: a rapid review. *Public Heal Res* 2018;**6**:1–162. doi:10.3310/phr06130
9 Morales ME, Berkowitz SA. The Relationship Between Food Insecurity, Dietary Patterns, and Obesity. *Curr Nutr Rep* 2016;:1–7. doi:10.1007/s13668-016-0153-y
10 Jyoti DF, Frongillo EA, Jones SJ. Food insecurity affects school children's academic performance, weight gain, and social skills. *J Nutr* 2005;**135**:2831–9. doi:10.1093/jn/135.12.2831
11 Whitaker RC, Phillips SM, Orzol SM. Food insecurity and the risks of depression and anxiety in mothers and behavior problems in their preschool-aged children. *Pediatrics* 2006;**118**:e859-68. doi:10.1542/peds.2006-0239
12 Molcho M, Gabhainn SN, Kelly C, *et al.* Food poverty and health among schoolchildren in Ireland: findings from the Health Behaviour in School-aged Children (HBSC) study. *Public Health Nutr* 2007;**10**:364–70. doi:10.1017/S1368980007226072
13 McLaughlin KA, Green JG, Alegría M, *et al.* Food insecurity and mental disorders in a national sample of U.S. adolescents. *J Am Acad Child Adolesc Psychiatry* 2012;**51**:1293–303. doi:10.1016/j.jaac.2012.09.009
14 Kimbro RT, Denney JT. Transitions Into Food Insecurity Associated With Behavioral Problems And Worse Overall Health Among Children. *Health Aff (Millwood)* 2015;**34**:1949–55. doi:10.1377/hlthaff.2015.0626
15 Poole-Di Salvo E, Silver EJ, Stein REK. Household Food Insecurity and Mental Health Problems Among Adolescents: What Do Parents Report? *Acad Pediatr* 2016;**16**:90–6. doi:10.1016/j.acap.2015.08.005
16 King C. Food insecurity and child behavior problems in fragile families. *Econ Hum Biol* 2018;**28**:14–22. doi:10.1016/j.ehb.2017.11.002
17 Alaimo K, Olson CM, Frongillo EA, *et al.* Food insufficiency, family income, and health in US preschool and school-aged children. *Am J Public Health* 2001;**91**:781–6. doi:10.2105/ajph.91.5.781
18 Thomas MMC, Miller DP, Morrissey TW. Food Insecurity and Child Health. *Pediatrics* 2019;**144**:e20190397. doi:10.1542/peds.2019-0397
19 Slopen N, Fitzmaurice G, Williams DR, *et al.* Poverty, food insecurity, and the behavior for childhood internalizing and externalizing disorders. *J Am Acad Child Adolesc Psychiatry* 2010;**49**:444–52. doi:10.1097/00004583-201005000-00005

For peer review only - http://bmjopen.bmj.com/site/about/guidelines.xhtml

20 Burke MP, Martini LH, Çayır E, *et al.* Severity of Household Food Insecurity Is Positively Associated with Mental Disorders among Children and Adolescents in the United States. *J Nutr* 2016;**146**:2019–26. doi:10.3945/jn.116.232298
21 The Food Foundation. A Crisis within a Crisis: The Impact of Covid-19 on Household Food Security. 2021. https://foodfoundation.org.uk/wp-content/uploads/2021/03/FF_Impact-of-Covid_FINAL.pdf
22 Bramley G, Treanor M, Sosenko F, *et al.* State of Hunger: Building the evidence on poverty, destitution, and food insecurity in the UK. 2021.
23 Gregory J, Lowe S, Bates C, *et al.* National Diet and Nutrition Survey: young people aged 4 to 18 years. Volume 1: report of the Diet and Nutrition Survey. 2000.
24 Stevens L, Nelson M. The contribution of school meals and packed lunch to food consumption and nutrient intakes in UK primary school children from a low income population. *J Hum Nutr Diet* 2011;**24**:223–32. doi:10.1111/j.1365-277X.2010.01148.x
25 Pike J, Colquhoun D. The relationship between policy and place: The role of school meals in addressing health inequalities. *Heal Sociol Rev* 2009;**18**:50–60. doi:10.5172/hesr.18.1.50
26 Taylor J, Garnett B, Horton MA, *et al.* Universal Free School Meal Programs in Vermont Show Multi-domain Benefits. *J Hunger Environ Nutr* 2020;**15**:753–66. doi:10.1080/19320248.2020.1727807
27 House of Commons Library, Long R, Danechi S, *et al.* School meals and nutritional standards (England). 2021.
28 Department of Education. Free school meals: Autumn term. 2021.https://explore-education-statistics.service.gov.uk/find-statistics/free-school-meals-autumn-term/2020-21-autumn-term (accessed 27 Sep 2021).
29 Sahota P, Woodward J, Molinari R, *et al.* Factors influencing take-up of free school meals in primary-and secondary-school children in England. *Public Health Nutr* 2014;**17**:1271–9. doi:10.1017/S136898001300092X
30 Blundell R, Costa Dias M, Joyce R, *et al.* COVID-19 and Inequalities*. *Fisc Stud* 2020;**41**:291–319. doi:10.1111/1475-5890.12232
31 Judge L, Rahman F. Lockdown living: Housing quality across the generations. Published Online First: 2020.https://www.resolutionfoundation.org/publications/lockdown-living/
32 Public Health England. Beyond the data : Understanding the impact of COVID-19 on BAME groups About Public Health England. 2020;:69.https://assets.publishing.service.gov.uk/government/uploads/system/uploads/attachment_data/file/892376/COVID_stakeholder_engagement_synthesis_beyond_the_data.pdf
33 Norman J. Gender and Covid-19: the immediate impact the crisis is having on women. *Br Policy Polit LSE* 2020;:1–5.http://eprints.lse.ac.uk/104638/
34 Windsor-Shellard B, Butt A. Coronavirus (COVID-19) related deaths by occupation, England and Wales. 2020.https://www.ons.gov.uk/peoplepopulationandcommunity/healthandsocialcare/causesofdeath/datasets/coronaviruscovid19relateddeathsbyoccupationenglandandwales (accessed 13 Dec 2020).
35 Read B, Low H, Jäckle A, *et al.* The idiosyncratic impact of an aggregate shock: the distributional consequences of COVID-19. 2020. doi:10.1920/wp.ifs.2020.1520
36 Marmot M, Allen J, Goldblatt P, *et al.* Build Back Fairer: The COVID-19 Marmot Review. The Pandemic, Socioeconomic and Health Inequalities in England. *Marmot Rev* 2020;:1–221.https://www.health.org.uk/publications/build-back-fairer-the-covid-19-marmot-review
37 Child Poverty Action Group. The cost of learning in lockdown: Family experiences of school closures. 2020.
38 Power M, Patrick R, Garthwaite K, *et al.* COVID realities - everyday life for families on a low income during the pandemic. 2020.
39 Brewer M, Patrick R. Pandemic Pressures: Why families on a low income are spending more

For peer review only

during Covid-19. 2021.
40 Patrick R, Anstey K, Lee T, *et al.* Fixing Lunch : The case for expanding free school meals. 2021;:1–14.
41 Melchior M, Chastang JF, Falissard B, *et al.* Food Insecurity and Children's Mental Health: A Prospective Birth Cohort Study. *PLoS One* 2012;**7**:1–7. doi:10.1371/journal.pone.0052615
42 R Core Team. R: A language and environment for statistical computing. 2020.https://www.r-project.org/
43 Bruening M, Lucio J. Parent-child relationships between food insecurity and eating behaviors. *FASEB J* 2015;**29**. doi:10.1096/fasebj.29.1_supplement.585.23
44 Tarasuk V, McIntyre L, Li J. Low-Income Women's Dietary Intakes Are Sensitive to the Depletion of Household Resources in One Month. *J Nutr* 2007;**137**:1980–7. doi:10.1093/jn/137.8.1980
45 Power M, Small N, Doherty B, *et al.* Hidden hunger? Experiences of food insecurity amongst Pakistani and white British women. *Br Food J* 2018;**120**:2716–32. doi:10.1108/BFJ-06-2018-0342
46 Defeyter G E a. Food And Coping Strategies during the Covid-19 Pandemic. *J Chem Inf Model* 2016;**53**:1689-1699Predicting the binding mode of flexible p.
47 Loopstra R, Reeves A, Tarasuk V. The rise of hunger among low-income households: An analysis of the risks of food insecurity between 2004 and 2016 in a population-based study of UK adults. *J Epidemiol Community Health* 2019;**73**:668–73. doi:10.1136/jech-2018-211194
48 Tingay RS, Tan CJ, Tan NCW, *et al.* Food insecurity and low income in an English inner city. *J Public Health Med* 2003;**25**:156–9. doi:10.1093/pubmed/fdg032
49 Loopstra R. Vulnerability of Food Insecurity since COVID-19 Lockdown. 2020.
50 Koltai J, Toffolutti V, McKee M, *et al.* Changing probability of experiencing food insecurity by socioeconomic and demographic groups during the COVID-19 pandemic in the UK. *medRxiv* 2020;:0–18.
51 Pybus K, Power M, Pickett KE. 'We are constantly overdrawn, despite not spending money on anything other than bills and food': a mixed-methods, participatory study of food and food insecurity in the context of income inequality. *J Poverty Soc Justice* 2020;**xx**:1–22. doi:10.1332/175982720x15998354133521
52 MacLeod MA, Curl A, Kearns A. Understanding the Prevalence and Drivers of Food Bank Use: Evidence from Deprived Communities in Glasgow. *Soc Policy Soc* 2019;**18**:67–86. doi:10.1017/S1474746418000064
53 Bazerghi C, McKay FH, Dunn M. The Role of Food Banks in Addressing Food Insecurity: A Systematic Review. *J Community Health* 2016;**41**:732–40. doi:10.1007/s10900-015-0147-5
54 Caplan P. Big society or broken society? *Anthropol Today* 2016;**21**:443–55.http://www.journals.cambridge.org/abstract_S1474746415000184
55 POWER M, DOHERTY B, SMALL N, *et al.* All in it Together? Community Food Aid in a Multi-Ethnic Context. *J Soc Policy* 2017;**46**:447–71. doi:10.1017/S0047279417000010
56 Melchior M, Caspi A, Howard LM, *et al.* Mental health context of food insecurity: a representative cohort of families with young children. *Pediatrics* 2009;**124**:e564-72. doi:10.1542/peds.2009-0583
57 Harwood E. Discretion, dignity and choice : free school meals. 2018;:2018–20.

For peer review only - http://bmjopen.bmj.com/site/about/guidelines.xhtml

Acknowledgements

We would like to take this opportunity to thank The Food Foundation and ChildWise for allowing us to conduct analyses on their COVID-19 survey data and to the children and families who took part in the surveys.

Funding

This work is supported in part by the FixOurFood programme (BB/V004581/1) funded by the UK Research and Innovation (UKRI) Transforming Food Systems Programme; ActEarly UK Prevention Research Partnership Consortium (MR/S037527/1); The National Institute for Health Research under its Applied Research Collaboration Yorkshire and Humber (NIHR200166); and The Health Foundation COVID-19 Award (2301201).

Competing interests
All authors have completed the ICMJE uniform disclosure form at www.icmje.org/disclosure-of-interest/ and declare: support from the Bradford Institute for Health Research and the University of York; no financial relationships with any organisations that might have an interest in the submitted work in the previous three years; no other relationships or activities that could appear to have influenced the submitted work.

Transparency statement

The corresponding author (TY) affirms that the manuscript is an honest, accurate, and transparent account of the study being reported. No important aspects of the study have been omitted and any discrepancies from the study as originally planned have been disclosed.

Dissemination to participants and related patient and public communities

Findings will be disseminated through media outreach to the general public.

Contributors
TCY, MP, RHM, MB designed and planned the study. MB acquired the data. TCY performed the statistical analysis and was responsible for the initial draft of the manuscript. All authors were involved in interpreting the study results, revising the manuscript, and approving the final version for submission. MB is the guarantor. The corresponding author attests that all listed authors meet authorship criteria and that no others meeting the criteria have been omitted.

Ethics approval
None required.

Data availability statement
The datasets analysed during the current study are not freely available. Applications to access the data can be made to The Food Foundation.

The Corresponding Author has the right to grant on behalf of all authors and does grant on behalf of all authors, a worldwide licence to the Publishers and its licensees in perpetuity, in all forms, formats and media (whether known now or created in the future), to i) publish, reproduce, distribute, display and store the Contribution, ii) translate the Contribution into other languages, create adaptations, reprints, include within collections and create summaries, extracts and/or, abstracts of the

For peer review only - http://bmjopen.bmj.com/site/about/guidelines.xhtml

Contribution, iii) create any other derivative work(s) based on the Contribution, iv) to exploit all subsidiary rights in the Contribution, v) the inclusion of electronic links from the Contribution to third party material where-ever it may be located; and, vi) licence any third party to do any or all of the above.

For peer review only

For peer review only - http://bmjopen.bmj.com/site/about/guidelines.xhtml

Table 1. Characteristics of the survey population.

	Total sample n=2166	
	N	Mean (SD)/%
**Parent age**		
18-24	8	0.4
25-34	268	12.4
35-44	923	42.6
45-54	762	35.2
55-64	205	9.5
**Parent occupation**		
Higher	1341	61.9
Lower	825	38.1
**Geographical region**		
East Midlands	158	7.3
Eastern	196	9
London	282	13
North East	92	4.2
North West	240	11.1
Northern Ireland	73	3.4
Scotland	161	7.4
South East	300	13.9
South West	197	9.1
Wales	109	5
West Midlands	182	8.4
Yorkshire & Humberside	176	8.1
**Number in household**		
2	160	7.4
3	624	28.8
4	939	43.4
5	318	14.7
6+	125	5.8
**Child's ethnicity**		
Asian	245	11.4
Other	209	9.7
White	1691	78.8
**Child's age**	2166	12.4 (3.2)
**Child sex**		
Female	1076	49.7
Male	1090	50.3
**Child receives FSM**		
Yes	675	31.5
No	1467	68.5
**Potential food insecurity**		
Yes	431	20.6
No	1659	79.4

For peer review only

**Any food bank use**		
Yes	561	25.9
No	1605	74.1
**Food insecure***		
Yes	763	35.2
No	1403	64.8

*Defined as responding affirmatively to any of the 6 potential food insecurity questions or indicated any food bank use

For peer review only

For peer review only - http://bmjopen.bmj.com/site/about/guidelines.xhtml

Table 2. Food insecurity and food bank use by children who receive or do not receive free school meals.

	Receives FSM					Does not receive FSM				
	Food insecurity n=407 (60%)		No food insecurity n=268 (40%)			Food insecurity n=338 (23%)		No food insecurity n=1129 (77%)		
	N	%	N	%	*p*-value*	N	%	N	%	*p*-value*
**Parent age**					<0.001					<0.001
18-24	5	1.2	0	0		1	0.3	2	0.2	
25-34	91	22.4	44	16.4		43	12.7	87	7.7	
35-44	194	47.7	115	42.9		167	49.4	436	38.6	
45-54	97	23.8	75	28		107	31.7	474	41.9	
55-64	20	4.9	34	12.7		20	5.9	130	11.5	
**Parent occupation**					<0.001					0.1
Higher	238	58	106	40		214	63	754	68	
Lower	169	42	162	60		124	37	364	32	
**Number in household**					0.6					0.2
2	37	9.1	29	10.8		25	7.4	63	5.6	
3	123	30.2	80	29.9		106	31.4	311	27.5	
4	164	40.3	95	35.4		142	42	527	46.7	
5	51	12.5	43	16		46	13.6	177	15.7	
6+	32	7.9	21	7.8		19	5.6	51	4.5	
**Child's ethnicity**					0.04					0.8
Asian	66	16	30	11		35	10.5	110	9.8	
Other	57	14	28	10		30	9	91	8.1	
White	282	70	210	78		268	80.5	916	82	

*$\chi 2$ or Fisher's exact test

For peer review only

Table 3. Odds of children reporting feeling stressed every day or most days in the past month.

	Unadjusted			Adjusted		
	N	OR	95% CI	N	OR	95% CI
Food insecurity*	1289	4.42	(3.29, 5.97)	1276	5.24	(3.84, 7.20)
Receives FSM**	1276	2.81	(2.10, 3.76)	1265	1.99	(1.42, 2.78)

*adjusted for child’s age and sex, ethnicity, parent occupation, household occupancy

**adjusted for child's age and sex, ethnicity, food insecurity, parent occupation, household occupancy

For peer review only

Figure 1. Percentage of children responding affirmatively to the six questions indicating potential food insecurity questions by FSM status.

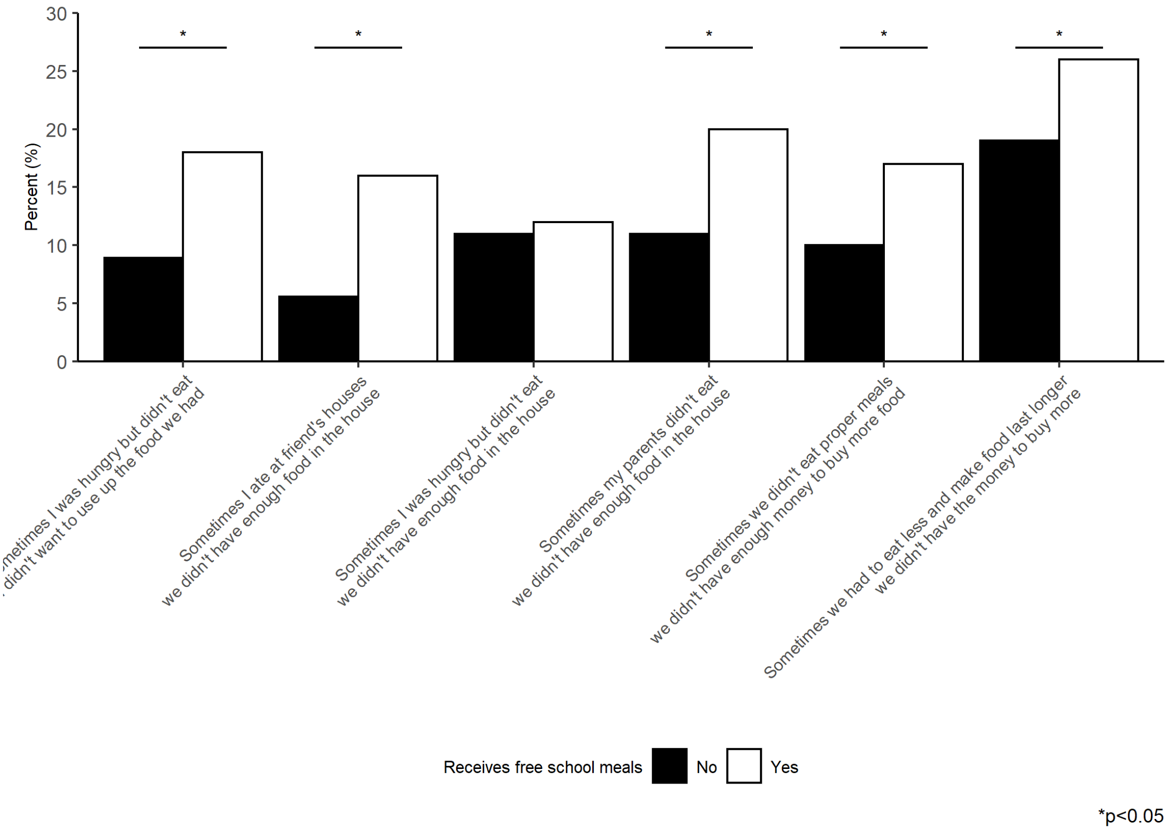

For peer review only - http://bmjopen.bmj.com/site/about/guidelines.xhtml

Figure 2. Percentage of children responding affirmatively to two questions indicating food bank use by FSM status.

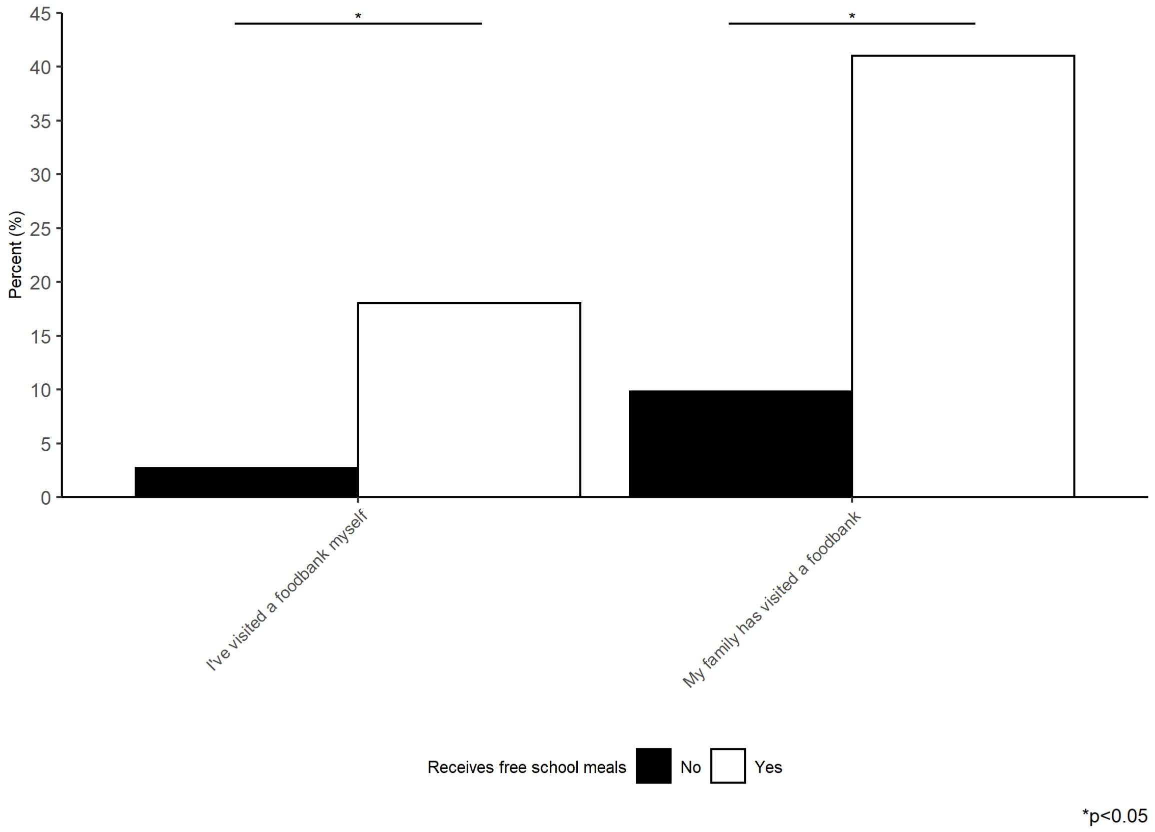

iew only

Figure 3. Percentage of children reporting sadness or worries in the past month according to FSM and food insecurity status.

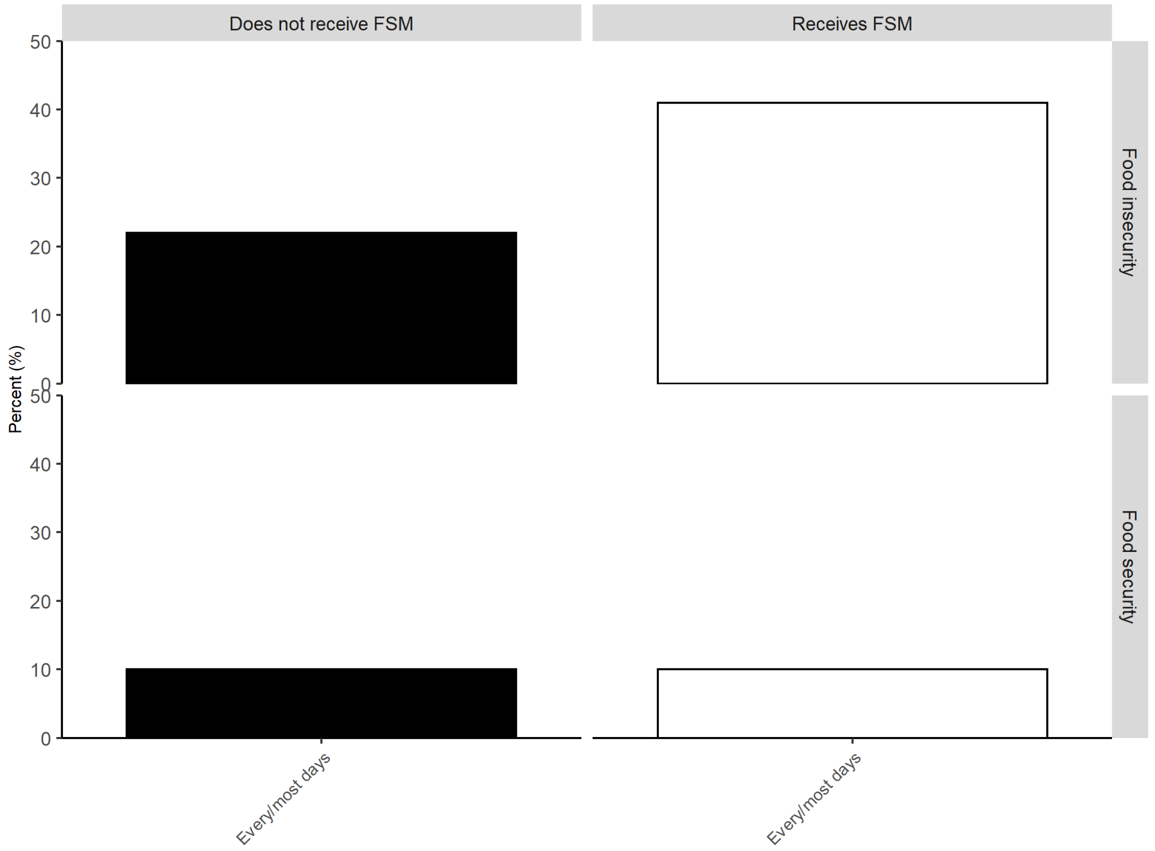

view only

Supplementary file 1. DAGs for (1) food insecurity and child mental health; (2) FSM and child mental health.

(1)

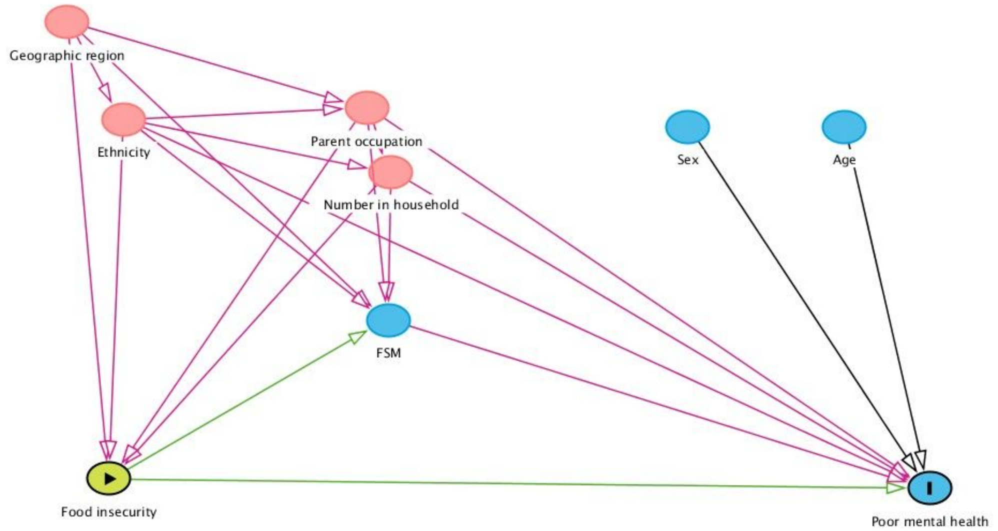

(2)

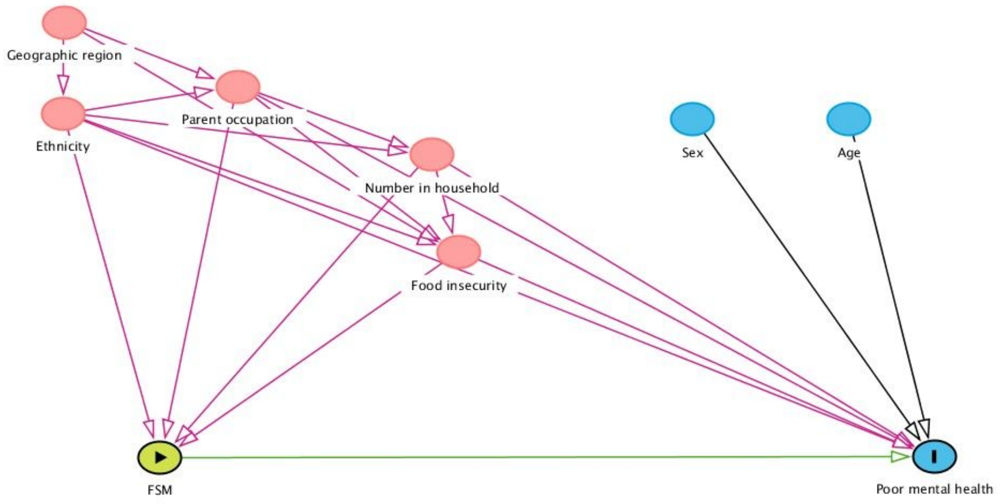

STROBE Statement—Checklist of items that should be included in reports of ***cross-sectional studies***

	Item No	Recommendation
**Title and abstract**	1	(*a*) Indicate the study's design with a commonly used term in the title or the abstract page 1
		(*b*) Provide in the abstract an informative and balanced summary of what was done and what was found page 1-2
**Introduction**		
Background/rationale	2	Explain the scientific background and rationale for the investigation being reported page 3-4
Objectives	3	State specific objectives, including any prespecified hypotheses page 4
**Methods**		
Study design	4	Present key elements of study design early in the paper page 4
Setting	5	Describe the setting, locations, and relevant dates, including periods of recruitment, exposure, follow-up, and data collection page 5
Participants	6	(*a*) Give the eligibility criteria, and the sources and methods of selection of participants page 4
Variables	7	Clearly define all outcomes, exposures, predictors, potential confounders, and effect modifiers. Give diagnostic criteria, if applicable pages 4-5
Data sources/ measurement	8*	For each variable of interest, give sources of data and details of methods of assessment (measurement). Describe comparability of assessment methods if there is more than one group pages 4-5
Bias	9	Describe any efforts to address potential sources of bias page 6
Study size	10	Explain how the study size was arrived at page 5
Quantitative variables	11	Explain how quantitative variables were handled in the analyses. If applicable, describe which groupings were chosen and why pages 4-6
Statistical methods	12	(*a*) Describe all statistical methods, including those used to control for confounding pages 5-6
		(*b*) Describe any methods used to examine subgroups and interactions pages 5-6
		(*c*) Explain how missing data were addressed page 5
		(*d*) If applicable, describe analytical methods taking account of sampling strategy pages 4-5
		(*e*) Describe any sensitivity analyses
**Results**		
Participants	13*	(a) Report numbers of individuals at each stage of study—eg numbers potentially eligible, examined for eligibility, confirmed eligible, included in the study, completing follow-up, and analysed page 4-5
		(b) Give reasons for non-participation at each stage page 4
		(c) Consider use of a flow diagram
Descriptive data	14*	(a) Give characteristics of study participants (eg demographic, clinical, social) and information on exposures and potential confounders page 6
		(b) Indicate number of participants with missing data for each variable of interest
Outcome data	15*	Report numbers of outcome events or summary measures page 6-7
Main results	16	(*a*) Give unadjusted estimates and, if applicable, confounder-adjusted estimates and their precision (eg, 95% confidence interval). Make clear which confounders were adjusted for and why they were included page 7
		(*b*) Report category boundaries when continuous variables were categorized

For peer review only

		(*c*) If relevant, consider translating estimates of relative risk into absolute risk for a meaningful time period
Other analyses	17	Report other analyses done—eg analyses of subgroups and interactions, and sensitivity analyses
**Discussion**		
Key results	18	Summarise key results with reference to study objectives pages 7-8
Limitations	19	Discuss limitations of the study, taking into account sources of potential bias or imprecision. Discuss both direction and magnitude of any potential bias page 9
Interpretation	20	Give a cautious overall interpretation of results considering objectives, limitations, multiplicity of analyses, results from similar studies, and other relevant evidence pages 7-9
Generalisability	21	Discuss the generalisability (external validity) of the study results
**Other information**		
Funding	22	Give the source of funding and the role of the funders for the present study and, if applicable, for the original study on which the present article is based page 14

*Give information separately for exposed and unexposed groups.

Note: An Explanation and Elaboration article discusses each checklist item and gives methodological background and published examples of transparent reporting. The STROBE checklist is best used in conjunction with this article (freely available on the Web sites of PLoS Medicine at http://www.plosmedicine.org/, Annals of Internal Medicine at http://www.annals.org/, and Epidemiology at http://www.epidem.com/). Information on the STROBE Initiative is available at www.strobe-statement.org.

For peer review only

For peer review only - http://bmjopen.bmj.com/site/about/guidelines.xhtml

BMJ Open

Are Free School Meals failing families? Exploring the relationship between child food insecurity, child mental health and Free School Meal status during COVID-19: national cross-sectional surveys

Journal:	*BMJ Open*
Manuscript ID	bmjopen-2021-059047.R1
Article Type:	Original research
Date Submitted by the Author:	11-Mar-2022
Complete List of Authors:	Yang, Tiffany; Bradford Teaching Hospitals NHS Foundation Trust<br>Power, Madeleine; University of York<br>Moss, Rachael ; Bradford Teaching Hospitals NHS Foundation Trust<br>Lockyer, Bridget; Bradford Institute for Health Research<br>Burton, Wendy; University of York<br>Doherty, Bob; University of York, The York Management School<br>Bryant, Maria; University of York, Health Sciences; Hull York Medical School
**Primary Subject Heading**:	Epidemiology
Secondary Subject Heading:	Health policy, Mental health, Nutrition and metabolism, Public health
Keywords:	MENTAL HEALTH, COVID-19, PUBLIC HEALTH

SCHOLARONE™
Manuscripts

I, the Submitting Author has the right to grant and does grant on behalf of all authors of the Work (as defined in the below author licence), an exclusive licence and/or a non-exclusive licence for contributions from authors who are: i) UK Crown employees; ii) where BMJ has agreed a CC-BY licence shall apply, and/or iii) in accordance with the terms applicable for US Federal Government officers or employees acting as part of their official duties; on a worldwide, perpetual, irrevocable, royalty-free basis to BMJ Publishing Group Ltd ("BMJ") its licensees and where the relevant Journal is co-owned by BMJ to the co-owners of the Journal, to publish the Work in this journal and any other BMJ products and to exploit all rights, as set out in our licence.

The Submitting Author accepts and understands that any supply made under these terms is made by BMJ to the Submitting Author unless you are acting as an employee on behalf of your employer or a postgraduate student of an affiliated institution which is paying any applicable article publishing charge ("APC") for Open Access articles. Where the Submitting Author wishes to make the Work available on an Open Access basis (and intends to pay the relevant APC), the terms of reuse of such Open Access shall be governed by a Creative Commons licence – details of these licences and which Creative Commons licence will apply to this Work are set out in our licence referred to above.

Other than as permitted in any relevant BMJ Author's Self Archiving Policies, I confirm this Work has not been accepted for publication elsewhere, is not being considered for publication elsewhere and does not duplicate material already published. I confirm all authors consent to publication of this Work and authorise the granting of this licence.

For peer review only

Are Free School Meals failing families? Exploring the relationship between child food insecurity, child mental health and Free School Meal status during COVID-19: national cross-sectional surveys

Tiffany C Yang[1] (0000-0003-4549-7850) Principal research fellow, Madeleine Power[2] (0000-0002-9571-1782) Senior research fellow, Rachael H Moss[1] (0000-0001-6160-8536) Research fellow, Bridget Lockyer[1] (0000-0002-2195-5549) Senior research fellow, Wendy Burton[2] (0000-0001-7885-5971) Research associate, Bob Doherty[3] (0000-0001-6724-7065) Professor, Maria Bryant[2,4] (0000-0001-7690-4098) Reader
[1]Bradford Institute of Health Research, Bradford Teaching Hospital NHS Foundation Trust, Bradford, BD9 6RJ, UK
[2]Department of Health Sciences, University of York, York, YO10 5DD, UK
[3]University of York Management School, University of York, York, YO10 5DD, UK
[4]Department of Health Sciences and the Hull York Medical School, University of York, York, YO10 5DD, UK

Correspondence to:
Tiffany Yang
Bradford Institute for Health Research
Bradford Royal Infirmary
Duckworth Lane
Bradford BD9 6RJ
Tiffany.yang@bthft.nhs.uk

Abstract

Objective: Food insecurity is linked to poor health and wellbeing in children, and rising prevalence rates have been exacerbated by COVID-19. Free School Meals (FSM) are considered a critical tool for reducing the adverse effects of poverty but apply a highly restrictive eligibility criteria. This study examined levels of food security and FSM status to support decision making regarding increasing the current eligibility criteria.

Design: Two cross-sectional national surveys administered in August-September 2020 and January-February 2021 were used to examine the impact of COVID-19 on the food experiences of children and young people.

Setting: United Kingdom.

Participants: 2,166 children (aged 7-17 years) and parents/guardians.

Main outcome measures: Participant characteristics were described by food security and FSM status; odds of poor mental health, reported as children reporting feeling stressed or worried in the past month, by food security and FSM status, adjusted for confounding variables.

Results: We observed food insecurity among both children who were or were not in receipt of FSM: 23% of children not receiving FSM were food insecure. Children who were food insecure had worse mental health compared to children who were food secure (Odds Ratio [OR]: 5.24, 95% Confidence Interval [95% CI]: 3.84 to 7.20); and this was lower among children receiving FSM (OR: 1.99, 95% CI: 1.42 to 2.78) compared to those who were not.

Conclusion: Many children experienced food insecurity regardless of whether they received FSM suggesting the eligibility criteria needs to be widened to prevent overlooking those in need.

For peer review only - http://bmjopen.bmj.com/site/about/guidelines.xhtml

Keywords

Food insecurity, child food insecurity, free school meals, mental health, COVID-19.

Strengths and limitations

- Geographic and demographically-representative sample with wide range of ages of children
- Child-reported measures of food insecurity and mental health
- Parents may have helped their child complete the survey and influenced responses among younger children
- Analyses were not stratified by age though food insecurity may be experienced differently between those of younger and older ages
- We were unable to differentiate between eligibility and uptake

For peer review only

Introduction

The past decade has witnessed sharp rises in the use of food banks by households with children, suggesting that child food insecurity is rising[1]. In that decade, the Trussell Trust reported a 31-fold increase in the number of emergency food parcels distributed, from 61,000 in 2010-2011 to 1.9 million in 2019/2020 [2]. Food insecurity can broadly be defined as uncertainty around the quality and quantity of food available [3]. Data from the Family Resource Survey (FRS) prior to the COVID-19 pandemic indicates that households with children are at particular risk of food insecurity in the UK [3–5]. In 2019/2020, five million people in the UK (8%) were in food insecure households, of whom 13% were children, 8% were working age adults, and 2% were pensioners [4]. Food insecurity has considerable nutritional, physical, and cognitive implications for children, including, but not limited to, associations with lower vegetable intake, higher added sugar intake [6,7], increased risk of obesity [5,8–10] and poorer academic performance [11,12]. There is a growing body of literature - almost entirely from North America - evidencing an association between the experience of food insecurity and an increase in the risk of mental health issues for children and adolescents [13–18]. Children and teenagers experiencing food insecurity report lower life satisfaction [14], and have a higher probability of seeing a psychologist and finding it difficult to make friends [19]. Evidence suggests that rates of depression [20], stress and anxiety are higher for children living in food insecure households [15,21,22]. Households with children have been particularly badly affected by the social and economic fallout of the COVID-19 pandemic. In the first six months of the pandemic, 12% of adults living with children reported skipping meals because they could not afford or access food, while 4% of adults with children reported going for a whole day without eating [23]. Food banks also reported a sharp rise in access by households with young children. Between early and mid-2020, The Trussell Trust food bank network supported 370,000 households, of which 320,000 were families with children. The proportion of couples with children referred to a food bank increased from 19% in early 2020 to 24% during the COVID-19 pandemic in mid-2020 [2].

Free school meals (FSM) are considered to be a critical tool for mitigating the negative health effects of child poverty among low-income families. Children receiving FSM obtain a higher proportion of their daily energy and nutrient intakes from their school meals compared to those who pay [24,25] and FSM may therefore improve health and wellbeing and reduce health inequalities [26,27]. In England, FSM are currently a statutory entitlement available to eligible pupils, which include all infant school children (reception, year 1, and year 2) in state-funded schools (as part of the Education Act, 1944) [28]; and pupils in year 3 and upwards (junior school and secondary school pupils) whose parents meet income-defined eligibility criteria[1] and claim for FSM. As of 1 October 2020, there were 1.63 million pupils known to be eligible for FSM, including those part of the universal FSM offer, [29], an increase in the proportion eligible to 19.7% of all state-funded pupils from 17.3% in January 2020 and 15.4% in January 2019. This increase is likely due to increased unemployment during the COVID-19 pandemic rendering more children eligible for FSM, alongside increased uptake due to greater media attention and awareness of FSM.

FSM receipt can be considered a marker of poverty due to its restrictive eligibility criteria and children who receive FSM are likely to be living in low income households. The COVID-19 pandemic

---

[1] Parents currently meet the eligibility criteria if they receive: Income Support; Income-based Jobseekers Allowance; Income-related Employment and Support Allowance; Support under Part VI of the Immigration and Asylum Act 1999; the guaranteed element of State Pension Credit; Child Tax Credit (provided they are not also entitled to Working Tax Credit and have an annual gross income of no more than £16,190); Working Tax Credit run-on (paid for four weeks after a person stops qualifying for Working Tax Credit); and Universal Credit (with household income of less than £7,400 a year after tax and not including any benefits).

For peer review only - http://bmjopen.bmj.com/site/about/guidelines.xhtml

has exposed and amplified pre-existing concerns about the restrictive eligibility criteria for FSM (for pupils above year 2) and low uptake of FSM among eligible families (both before and after registration)[30]. The effects of the pandemic have been highly unequal, according to income, ethnicity, gender and health status [31–37]. There is evidence to suggest that low income families have been particularly negatively affected by the social and economic circumstances of the pandemic [38–40] and yet have thus far been largely neglected in the Governmental policy response. Emerging evidence suggests that families just outside of the eligibility criteria for FSM have struggled to afford food during the pandemic, potentially exacerbating child food insecurity [41]. However, this has not yet been formally assessed. This paper addresses this important and urgent research gap, examining the relationship between child food insecurity and families in receipt of and not in receipt of FSM during the pandemic. Given the known negative effects of food insecurity on child mental health and educational outcomes [11,13,42], the paper also looks at child mental health in the context of child food insecurity and receipt of FSM.

Methods

Study population and survey design

Data were taken from two Food Foundation commissioned surveys (ChildWise) conducted in the course of the COVID-19 pandemic to examine its impact on children and young people's COVID-19 food experiences. The first (August-September 2020; response rate: 10%) and second surveys (January-February 2021; response rate: 28%) were carried out online using a carefully constructed framework to ensure a geographic and demographic representative sample of adults living in the UK with children and young people aged 7-17 years. Children younger than 7 years of age, including children aged 7 in Year 2 of primary school, were excluded in order to capture children's experiences outside of universal FSM provision.

The online panel used by ChildWise is a member of the ESOMAR (European Society of Opinion and Marketing Research) organisation and endeavours to be as representative as possible. This panel is the largest in the UK and globally. To achieve representative quotas, the panel's profiling data was first used to target the more difficult-to-reach demographics before targeting other groups. Samples were recruited to be representative by region, broad ethnic group, and spread evenly by age and gender.

Surveys were completed by parents or guardians (hereafter "parents") of children with a section to be completed by children with the aid of parents if required. Parents were asked to list the ages and genders of all children in the household and one child was initially randomly allocated to complete the child portion of the survey. Parents completed questions on socio-demographic details and were asked to complete information about up to two of their children's FSM status, age, and gender. Children completed questions on perception of FSM, food insecurity and food bank use. In the second survey, additional questions on the child's mental health were included in the children's section. Towards the end of the fieldwork period, children were non-randomly assigned to complete the child portion of the survey based on fulfilling any remaining quotas of age, gender, and geographic region.

Patient and public involvement

The survey used in this study was developed in partnership with Food Foundation, who have established a group of young food ambassadors to help set priority areas. This group meets on a regular basis to discuss important and immerging areas of interest that have the best chance of policy change. They have provided advice on asking questions to young people and the methodological approach used.

For peer review only - http://bmjopen.bmj.com/site/about/guidelines.xhtml

Sociodemographic characteristics

Parents completed questions on their and their child's age and gender, their child, their ethnicity and occupation of the Chief Income Earner. We collapsed ethnicity from 12 categories into three: White (White British; Other White Background); Asian (Bangladeshi; Chinese; Indian; Pakistani; Other Asian Background) and Other (Black African; Black Caribbean; Other Black Background; Mixed background). Participants who chose "Prefer not to answer" were coded to missing.

Parental occupation was reported for the Chief Income Earner in the household, defined as the individual within the household with the largest income. If the Chief Income Earner was not in paid employment but has been out of work for fewer than 6 months, the most recent occupation was reported. If the Chief Income Earner was retired with an occupation pension, then the most recent occupation was reported. Twelve categories of occupations were collapsed by ChildWise into two categories of social grade (ABC1 and C2DE), with the higher occupational class as a shorthand for middle class (ABC1) and the lower occupational class as shorthand for working class (C2DE): Higher (Supervisory or clerical/ junior managerial/ professional/ administrative; Intermediate managerial/ professional/ administrative; Higher managerial/ professional/ administrative; Student) and Lower (Semi or unskilled manual work; Skilled manual worker; Casual worker - not in permanent employment; Housewife/Homemaker; Retired and living on state pension; Unemployed or not working due to long-term sickness; Full-time carer of other household member; Other).

Free School Meals

Parents were asked whether their child was currently registered for FSM. Responses were coded to "Yes" if parents responded "Yes" and "No" if parents responded "No". Responses of "Don't know" and "Prefer not to say" were coded to missing. Responses were similar to the question asked of children ("Thinking about when you have lunch at school, do you have Free School Meals?").

Food insecurity

Children were asked to think about being at home during the summer holidays (August-September 2020 survey) or the Christmas holidays and recent lockdown (January-February 2021 survey) and asked to respond to several statements regarding potential food insecurity. Children were categorised as having "potential food insecurity" if they responded "Yes" to any of the following 6 statements: (1) Sometimes I was hungry but didn't eat because I didn't want to use up the food we had; (2) Sometimes I was hungry but didn't eat because we didn't have enough food in the house; (3) Sometimes my parents didn't eat because we didn't have enough food in the house; (4) Sometimes we had to eat less and make food last longer because we didn't have the money to buy more; (5) Sometimes we didn't eat proper meals because we didn't have enough money to buy more food; (6) Sometimes I ate at friend's houses because we didn't have enough food in the house. Children were categorised as not having potential food insecurity if they responded "Yes" to "I always had enough food to eat". There were no children who responded affirmatively to both "I always had enough to eat" and any of the other 6 statements. Children who responded affirmatively to "Don't know" or "Prefer not to say" were coded to missing.

Children were also asked to respond to several statements regarding food bank use. They were coded to any food bank use if they responded "Yes" to having visited a food bank by themselves or if their family if they responded "No" to the statement "No, we didn't visit a food bank". Remaining children were coded as not having used a food bank.

A dichotomous variable of "food insecurity" was then generated and included children who were identified as having "potential food insecurity" (from the 6 questions) or indicated any food bank use. Children who did not have "potential food insecurity" and did not indicate any food bank use were considered to be "food secure". Children who responded "yes" to any of the "potential food insecurity" questions or indicated that they or their family had visited a food were considered to be "food insecure".

For peer review only - http://bmjopen.bmj.com/site/about/guidelines.xhtml

Mental health

Among children who responded affirmatively that they received FSM, they were asked to select from a range of statements on how they felt about FSM. We examined their responses to "I think it is embarrassing to have free school meals"; affirmative responses were coded to "Yes, embarrassed" and negative responses were coded to "No, not embarrassed".

Children participating in the January-February 2021 survey were asked how often they felt stressed or worried in the past month and were categorised as being stressed or worried "Every/most days" if they said they were worried "every day" or "most days". Children were categorised as being stressed or worried "Some/rarely" if they said they were worried "Some days", "Rarely", or "I have not felt stressed once in the last month".

Data analysis

All analyses were conducted in R version 4.0.2[43]. Responses were combined and analysed across both surveys. We examined differences in characteristics by survey period and did not find differences by measures of food insecurity or receipt of FSM (Supplementary file 1). A small number of participants responded to both surveys (n=206). Their responses were removed from the first survey so that they were present in the sample only once and were able to be part of the mental health analysis. Participants who responded to both surveys were less likely to have used a food bank or be food insecure (Supplementary file 2). Main analyses were completed on a sample size of n=2,166.

Responses were described using mean (standard deviation [SD]) for continuous measures and number (n) and percentage (%) for categorical measures. We constructed 4 groups: (1) children with food insecurity who had FSM; (2) children with food insecurity who did not have FSM; (3) children without food insecurity who had FSM; (4) children without food insecurity who did not have FSM.

Differences between participant characteristics and responses to food insecurity questions, food bank use, and derived food insecurity by children who received or did not receive FSM were assessed using χ^2 or Fisher's exact tests for categorical variables and Welch's two-sample t-test for continuous variables. A significant p-value (p<0.05) indicates that there is a difference between the characteristics by food security status among children who receive FSM and among children who do not receive FSM. In the subset of survey questions on children's mental health, unadjusted and adjusted logistic regression using complete case analysis were run and estimated marginal means obtained. Fully-adjusted analyses were performed with n=1,265 participants. A directed acyclic diagram (DAG) was drawn to assist in the selection of covariates (Supplementary file 3). In fully-adjusted analyses of food insecurity with child mental health, we included child age and sex, child ethnicity, parent occupation, household occupancy, region, receipt of FSM, and an interaction term between food insecurity and FSM. We calculated the probability of our outcome for every combination of food security status and FSM status while holding all covariates at their mean or mode using the "predictions" function in the "marginal effects" package.

Results

Participant characteristics

The majority (78%) of parent respondents were aged 35-54 years old, were professionally employed (61.9%), and lived in households with two or three people (72%) (Table 1). The majority of children were White (78.8%) or Asian (11.4%) and female (57.2%) and just under a third of parents reported that their child received FSM (32%). Over 20% of children reported food insecurity, based on positively responding to any measure, and over a quarter reported that they or their family had visited a food bank (25.9%), placing over a third of children living with food insecurity according to our definition (35%). Among children who affirmed that they received FSM, a tenth (9.7%) expressed

For peer review only - http://bmjopen.bmj.com/site/about/guidelines.xhtml

that receiving FSM is embarrassing. Almost a fifth (18%) of children responding to the January-February 2021 survey reported that they felt stressed or worried every day or most days.

Among children who receive FSM, 60% were considered to have food insecurity (Table 2). Parents of children receiving FSM were younger, were more likely to be in a higher level of occupation, and less likely to be of White ethnicity. Over 20%of children who did not receive FSM reported food insecurity. Parents of children who did not receive FSM and had food insecurity were more likely to be younger than those not living with food insecurity. There was no difference in parental occupation between children who did not receive FSM and who lived with or without food insecurity with parents in both groups more likely to have a higher level of occupation. Children who were food insecure and who were in receipt of FSM were more likely to express that receiving FSM is embarrassing (13.1% compared to those who were not in receipt of FSM (4.5%; p<0.001). Children experiencing food insecurity were more likely to report feeling stress or worried every day or most days and this was greater among children in receipt of FSM than not.

Free school meals and potential food insecurity

More children receiving FSM reported any potential food insecurity measure than those not receiving FSM (42.8% among those who received FSM, 9.8% among those who did not; $p<0.05$). Figure 1 shows the percentage of children responding affirmatively to each of the 6 potential food insecurity questions by FSM status. Children receiving FSM were more likely to respond affirmatively to these questions, though many who did not receive FSM also indicated potential food insecurity. Among all children, the most commonly chosen item reported was having to eat less in order to make food last longer due to a lack of money to buy more food.

Free school meals and food bank use

Both children who did and did not receive FSM reported visiting a food bank, whether by themselves or their family (Figure 2). Children who received FSM were more likely to have reported visiting a food bank by themselves or their family than children not receiving FSM. We found 2.7% of children not receiving FSM visited a food bank by themselves while 9.8% reported their families had visited a foodbank.

Child's mental health

The probability of a child reporting being stressed or worried every day or most days was 31% among those reporting food insecurity and 10% among those not reporting food insecurity, adjusting for confounders (Figure 3). In models additionally examining FSM, the probability of a child reporting being stressed or worried every day was 51% among children with food insecurity and in receipt of FSM (Figure 4). Among children with food insecurity but not in receipt of FSM, the probability was 29%.

Discussion

In this family-based survey measuring experiences of the COVID-19 pandemic, we found a substantial number of children experienced food insecurity (defined here as having ever visited a food bank or experienced any food insecurity measure) regardless of whether they were in receipt of FSM. Food insecurity and measures of potential food insecurity were highest among children in receipt of FSM, likely reflecting the very low income threshold for FSM (currently £7,400), meaning that outside of universal infant provision (in England and Scotland), it is largely children in the very poorest families who receive FSM. In a subset of children with mental health measures, we found

For peer review only - http://bmjopen.bmj.com/site/about/guidelines.xhtml

that children who experienced food insecurity were more likely to report feeling stressed or worried on an almost-daily basis in the previous month compared to children who were food secure.

Children are often protected from hunger in families that experience food insecurity where parents report decreasing their own intake to shield their children [44–47]. We found that children reported their parents skipping meals due to a lack of food in the house. However, it was concerning that we also found children reporting hunger due to not having enough food in the house and almost 20%of all children living with food insecurity reporting having to eat less to make food stretch. The high proportion of children with food insecurity reporting food bank use is consistent with other reports that have highlighted the impact of the pandemic on levels of food insecurity [1]. The consequences of the pandemic on financial stability and, therefore, food insecurity, have also impacted families who may not have been previously affected.

Studies have observed food insecurity among the employed and recent data in the UK Longitudinal Household Study on food insecurity during the pandemic suggests that, while risk of food insecurity increased more for those who were unemployed, those who were persistently employed were also at risk [48–51]. The pandemic has exposed the notion that food insecurity occurs primarily among the unemployed or those less-skilled professions; over 50% of children reporting food insecurity in our data had parents with higher/professional levels of occupation. Educational attainment and income are not necessarily protective of food insecurity. In the 2019/2020 FRS, it was reported that 8% of households where the head of household obtained A-levels or Scottish Highers and 4% of households with further education and university qualifications were food insecure [4]. Likewise, increasing income increased the food security but even among those with a gross weekly income of £1,000 or more only 96% had high food security (meaning 4% experienced food insecurity), compared to 74% of households with a total gross weekly income of less than £200 have high food security.

Over 25% of all children and almost 50% of children living with food insecurity reported their families visiting a food bank. Previous research has suggested that use of food banks by UK households experiencing food insecurity is low [52,53]; however, our study suggests that, in the context of the pandemic, food bank use may have become more common for families experiencing food poverty. Food banks are a short-term 'emergency' response and concerns have been raised about the nutritional quality [54,55] and cultural adequacy of the food provided [56]. The emergence and continuation of food banks and the growing number of food parcels they provide may be seen as an example of 'successful' self-organisation around a need and conveys a sense that something is being done; however, it should be questioned whether it is the responsibility of the voluntary sector, rather than the Government, to provide access to something as basic as having food of sufficient quality and quantity. Food banks are often unable to provide fresh foods or ensure dietary requirements are met; continued reliance and widespread use of food banks, particularly among households with children, raises concerns about the long-term mental and physical implications for families relying on this form of 'emergency' support.

FSM are often seen as an essential tool for mitigating the effects of poverty experienced by children but provision is not universal or standardised across all nations, leading to unequal access (see Patrick et al., 2021). Our data reported more children registered for FSM (32%) compared to those reported eligible in autumn 2020 (19.7%) [29]. This may be reflected by an increase in newly-qualifying children for FSM as families lost income; in the first survey, over 40% of children registered for FSM had only recently started receiving FSM (i.e., were newly eligible that term) [23]. Once children age out of universal provision, stringent criteria must be met for children to receive FSM with many of the criteria being income-based, leading to only very low-income families being eligible and many low-income families going hungry. The eligibility is so restrictive that in our sample

For peer review only - http://bmjopen.bmj.com/site/about/guidelines.xhtml

nearly half of families who are food insecure do not receive FSM; the eligibility threshold is set at an annual household income of less than £7,400 prior to benefits while parents receiving Working Tax Credit are ineligible for FSM support regardless of their level of income. However, as we have shown, a large proportion of children experiencing food insecurity as well as those in receipt of FSM have parents employed in professional-level occupations. This suggests that the financial circumstances of families of all income levels have been hard-hit by the pandemic and that the current criteria may not be suitable for assessing eligibility. In addition, our findings that children who were receiving FSM still reported hunger suggests that FSM provision may not be sufficient to ensure that children are adequately fed on a daily basis.

There is limited published research in the UK on the role food insecurity plays in children's mental health, and none on the role of FSM in mitigating the association between food insecurity and poor mental health among children. Emerging UK evidence suggests poorer wellbeing and increased emotional and behavioural problems among children who experience food insecurity. One UK study found 27% of 10-year old children experiencing food insecurity exhibited clinically significant behavioural problems compared to 10% of children who were food secure [57]. Our findings that children who experience food insecurity have worse mental health are therefore unsurprising, and in line with North American literature on food insecurity and child mental health [13–18]. We found that children who reported food insecurity and received FSM had a higher probability of reporting feeling stressed or worried compared to children who did not receive FSM. This could potentially reflect the complex poverty-related stressors of living in a household eligible for FSM, and could indicate a more severe level of socioeconomic deprivation among children reporting food insecurity and receiving FSM, as well as the perceived stigma of receiving FSM [38,58]. Children who received FSM in this survey were asked whether they think it is embarrassing to have FSM and 11.5% of children living with food insecurity thought it was embarrassing compared to 4.1% of children who were food secure. This suggests children may carry an additional burden of stigma on top of inadequate food security.

There are multiple strengths and limitations to this study. This study used a geographic and demographically-representative sample with a wide range of ages and included measures of child-reported food insecurity combined with child reported mental health. However, we were unable to understand the degree to which parents may have helped their child complete the questions, and whether responses were given by them or were changed/given by their parents. This is more likely to have influenced responses from the younger children completing the survey. While the online panel used by ChildWise aims to be as representative as possible across geographic and demographic characteristics, it is possible that families within these representative categories who were more interested in the scope of the survey or who were food insecure would have participated, potentially skewing the responses towards those who experienced food insecurity or received FSM. Other studies conducted during the COVID-19 pandemic have also reported high prevalence of food insecurity, with 16.2% of adults surveyed during the first lockdown reporting experiencing food insecurity while the Understanding COVID-19 longitudinal study survey found the prevalence of food insecurity rose from 7.1% in April 2020 to 20.2% by July 2020 [50,51]. We also combined responses across two surveys and observed that there were fewer parents in the higher occupation category at the second survey (60%) compared to the first survey (65%) but did not find any differences in food insecurity, food bank use, or FSM when we examined participant characteristics by survey, suggesting it was appropriate to combine surveys. As some respondents participated in both surveys, we removed them from the August-September 2020 survey and included them in the January-February 2021 survey in order to maximize sample size for the logistic regression analyses; we examined whether participants who responded to both surveys were different from those responding to only the August-September 2020 survey and found that they were less likely to have visited a food bank or report food insecurity. Removing these participants

For peer review only - http://bmjopen.bmj.com/site/about/guidelines.xhtml

from the analyses did not alter our results. We were unable to explore experiences by more granular categories of ethnicity given small sample sizes among, for example, children of Black ethnicity, preventing examination of how other prevalent ethnic groups experience food insecurity. Similarly, categorisation of occupation into two groups may mask differences between occupations within each group and we only had occupation data for the Chief Income Earner, which may not adequately reflect the socioeconomic position of the household. We were additionally unable to examine the experiences of children who identify outside of the male-female binary as this information was not collected. The questions used to assess food insecurity and child's mental health were not from a standardised tool and therefore have not been validated; however, these questions still provide insight into the disruption of quality or quantity of food available and state of mind. We also did not stratify the sample to distinguish between children in primary or secondary school, which may reflect differences in how food insecurity is experienced, such as visiting a food bank themselves, as well as differential uptake in FSM regardless of eligibility. We were also unable to differentiate between eligibility and uptake and whether the 23% of children experiencing food insecurity but not receiving FSM were due to non-eligibility or from voluntary refusal as a result of stigma or other reasons for not participating when eligible, such as navigating the application process. Finally, due to the lack of information about parental income and other household financial constraints/resources, it is important to acknowledge that other than food insecurity and eligibility for FSM, we have not been able to identify any other factors (perhaps correlated with food insecurity) that may be impacting upon a child's mental health. Future research should consider other factors such as parental income, household income and receipt of benefits to help provide a more descriptive and causal picture of the financial status of participants' households.

Our findings confirm a real need to reconsider the eligibility criteria currently set for the provision of FSM. A concerning number of children are experiencing food insecurity in families with higher/professional levels of education who are likely to be above the eligibility threshold for FSM. While more families can be helped by widening eligibility, and more work is needed to understand access and uptake of FSM, including potential barriers such as social shame, policies which provide universal coverage should be considered as the impact goes beyond food provision and eliminates the stigma that is associated with being eligible and receiving FSM.

For peer review only

References

1 Power M, Doherty B, Pybus K, *et al.* How Covid-19 has exposed inequalities in the UK food system: The case of UK food and poverty. *Emerald Open Res* 2020;**2**:11. doi:10.35241/emeraldopenres.13539.1

2 Bramley G, Treanor M, Sosenko F, *et al.* State of Hunger: Building the evidence on poverty, destitution, and food insecurity in the UK. 2021.

3 Bickel G, Nord M, Price C, *et al.* Guide to Measuring Household Food Security. 2000.

4 Department of Work and Pensions. Family Resources Survey: financial year 2019 to 2020. 2021. https://www.gov.uk/government/statistics/family-resources-survey-financial-year-2019-to-2020/family-resources-survey-financial-year-2019-to-2020

5 Taylor A, Loopstra R. Too Poor to Eat Food insecurity in the UK. *foodfoundation.org.uk* 2016;:1–12.www.foodfoundation.org.uk

6 Eicher-Miller HA, Zhao Y. Evidence for the age-specific relationship of food insecurity and key dietary outcomes among US children and adolescents. *Nutr Res Rev* 2018;**31**:98–113. doi:10.1017/S0954422417000245

7 Hanson KL, Connor LM. Food insecurity and dietary quality in US adults and children: a systematic review. *Am J Clin Nutr* 2014;**100**:684–92. doi:10.3945/ajcn.114.084525

8 Yang TC, Sahota P, Pickett KE, *et al.* Association of food security status with overweight and dietary intake: exploration of White British and Pakistani-origin families in the Born in Bradford cohort. *Nutr J* 2018;**17**:48. doi:10.1186/s12937-018-0349-7

9 Aceves-Martins M, Cruickshank M, Fraser C, *et al.* Child food insecurity in the UK: a rapid review. *Public Heal Res* 2018;**6**:1–162. doi:10.3310/phr06130

10 Morales ME, Berkowitz SA. The Relationship Between Food Insecurity, Dietary Patterns, and Obesity. *Curr Nutr Rep* 2016;:1–7. doi:10.1007/s13668-016-0153-y

11 Jyoti DF, Frongillo EA, Jones SJ. Food insecurity affects school children's academic performance, weight gain, and social skills. *J Nutr* 2005;**135**:2831–9. doi:10.1093/jn/135.12.2831

12 Cohen JFW, Hecht AA, McLoughlin GM, *et al.* Universal school meals and associations with student participation, attendance, academic performance, diet quality, food security, and body mass index: A systematic review. *Nutrients* 2021;**13**:1–41. doi:10.3390/nu13030911

13 Whitaker RC, Phillips SM, Orzol SM. Food insecurity and the risks of depression and anxiety in mothers and behavior problems in their preschool-aged children. *Pediatrics* 2006;**118**:e859-68. doi:10.1542/peds.2006-0239

14 Molcho M, Gabhainn SN, Kelly C, *et al.* Food poverty and health among schoolchildren in Ireland: findings from the Health Behaviour in School-aged Children (HBSC) study. *Public Health Nutr* 2007;**10**:364–70. doi:10.1017/S1368980007226072

15 McLaughlin KA, Green JG, Alegría M, *et al.* Food insecurity and mental disorders in a national sample of U.S. adolescents. *J Am Acad Child Adolesc Psychiatry* 2012;**51**:1293–303. doi:10.1016/j.jaac.2012.09.009

16 Kimbro RT, Denney JT. Transitions Into Food Insecurity Associated With Behavioral Problems And Worse Overall Health Among Children. *Health Aff (Millwood)* 2015;**34**:1949–55. doi:10.1377/hlthaff.2015.0626

17 Poole-Di Salvo E, Silver EJ, Stein REK. Household Food Insecurity and Mental Health Problems Among Adolescents: What Do Parents Report? *Acad Pediatr* 2016;**16**:90–6. doi:10.1016/j.acap.2015.08.005

18 King C. Food insecurity and child behavior problems in fragile families. *Econ Hum Biol* 2018;**28**:14–22. doi:10.1016/j.ehb.2017.11.002

19 Alaimo K, Olson CM, Frongillo EA, *et al.* Food insufficiency, family income, and health in US preschool and school-aged children. *Am J Public Health* 2001;**91**:781–6. doi:10.2105/ajph.91.5.781

For peer review only - http://bmjopen.bmj.com/site/about/guidelines.xhtml

20 Thomas MMC, Miller DP, Morrissey TW. Food Insecurity and Child Health. *Pediatrics* 2019;**144**:e20190397. doi:10.1542/peds.2019-0397
21 Slopen N, Fitzmaurice G, Williams DR, *et al.* Poverty, food insecurity, and the behavior for childhood internalizing and externalizing disorders. *J Am Acad Child Adolesc Psychiatry* 2010;**49**:444–52. doi:10.1097/00004583-201005000-00005
22 Burke MP, Martini LH, Çayır E, *et al.* Severity of Household Food Insecurity Is Positively Associated with Mental Disorders among Children and Adolescents in the United States. *J Nutr* 2016;**146**:2019–26. doi:10.3945/jn.116.232298
23 The Food Foundation. A Crisis within a Crisis: The Impact of Covid-19 on Household Food Security. 2021. https://foodfoundation.org.uk/wp-content/uploads/2021/03/FF_Impact-of-Covid_FINAL.pdf
24 Gregory J, Lowe S, Bates C, *et al.* National Diet and Nutrition Survey: young people aged 4 to 18 years. Volume 1: report of the Diet and Nutrition Survey. 2000.
25 Stevens L, Nelson M. The contribution of school meals and packed lunch to food consumption and nutrient intakes in UK primary school children from a low income population. *J Hum Nutr Diet* 2011;**24**:223–32. doi:10.1111/j.1365-277X.2010.01148.x
26 Pike J, Colquhoun D. The relationship between policy and place: The role of school meals in addressing health inequalities. *Heal Sociol Rev* 2009;**18**:50–60. doi:10.5172/hesr.18.1.50
27 Taylor J, Garnett B, Horton MA, *et al.* Universal Free School Meal Programs in Vermont Show Multi-domain Benefits. *J Hunger Environ Nutr* 2020;**15**:753–66. doi:10.1080/19320248.2020.1727807
28 House of Commons Library, Long R, Danechi S, *et al.* School meals and nutritional standards (England). 2021.
29 Department of Education. Free school meals: Autumn term. 2021.https://explore-education-statistics.service.gov.uk/find-statistics/free-school-meals-autumn-term/2020-21-autumn-term (accessed 27 Sep 2021).
30 Sahota P, Woodward J, Molinari R, *et al.* Factors influencing take-up of free school meals in primary-and secondary-school children in England. *Public Health Nutr* 2014;**17**:1271–9. doi:10.1017/S136898001300092X
31 Blundell R, Costa Dias M, Joyce R, *et al.* COVID-19 and Inequalities*. *Fisc Stud* 2020;**41**:291–319. doi:10.1111/1475-5890.12232
32 Judge L, Rahman F. Lockdown living: Housing quality across the generations. Published Online First: 2020.https://www.resolutionfoundation.org/publications/lockdown-living/
33 Public Health England. Beyond the data : Understanding the impact of COVID-19 on BAME groups About Public Health England. 2020;:69.https://assets.publishing.service.gov.uk/government/uploads/system/uploads/attachment_data/file/892376/COVID_stakeholder_engagement_synthesis_beyond_the_data.pdf
34 Norman J. Gender and Covid-19: the immediate impact the crisis is having on women. *Br Policy Polit LSE* 2020;:1–5.http://eprints.lse.ac.uk/104638/
35 Windsor-Shellard B, Butt A. Coronavirus (COVID-19) related deaths by occupation, England and Wales. 2020.https://www.ons.gov.uk/peoplepopulationandcommunity/healthandsocialcare/causesofdeath/datasets/coronaviruscovid19relateddeathsbyoccupationenglandandwales (accessed 13 Dec 2020).
36 Read B, Low H, Jäckle A, *et al.* The idiosyncratic impact of an aggregate shock: the distributional consequences of COVID-19. 2020. doi:10.1920/wp.ifs.2020.1520
37 Marmot M, Allen J, Goldblatt P, *et al.* Build Back Fairer: The COVID-19 Marmot Review. The Pandemic, Socioeconomic and Health Inequalities in England. *Marmot Rev* 2020;:1–221.https://www.health.org.uk/publications/build-back-fairer-the-covid-19-marmot-review
38 Child Poverty Action Group. The cost of learning in lockdown: Family experiences of school closures. 2020.

For peer review only - http://bmjopen.bmj.com/site/about/guidelines.xhtml

39 Power M, Patrick R, Garthwaite K, *et al.* COVID realities - everyday life for families on a low income during the pandemic. 2020.

40 Brewer M, Patrick R. Pandemic Pressures: Why families on a low income are spending more during Covid-19. 2021.

41 Patrick R, Anstey K, Lee T, *et al.* Fixing Lunch : The case for expanding free school meals. 2021;:1–14.

42 Melchior M, Chastang JF, Falissard B, *et al.* Food Insecurity and Children's Mental Health: A Prospective Birth Cohort Study. *PLoS One* 2012;**7**:1–7. doi:10.1371/journal.pone.0052615

43 R Core Team. R: A language and environment for statistical computing. 2020.https://www.r-project.org/

44 Bruening M, Lucio J. Parent-child relationships between food insecurity and eating behaviors. *FASEB J* 2015;**29**. doi:10.1096/fasebj.29.1_supplement.585.23

45 Tarasuk V, McIntyre L, Li J. Low-Income Women's Dietary Intakes Are Sensitive to the Depletion of Household Resources in One Month. *J Nutr* 2007;**137**:1980–7. doi:10.1093/jn/137.8.1980

46 Power M, Small N, Doherty B, *et al.* Hidden hunger? Experiences of food insecurity amongst Pakistani and white British women. *Br Food J* 2018;**120**:2716–32. doi:10.1108/BFJ-06-2018-0342

47 Defeyter G E a. Food And Coping Strategies during the Covid-19 Pandemic. *J Chem Inf Model* 2016;**53**:1689-1699Predicting the binding mode of flexible p.

48 Loopstra R, Reeves A, Tarasuk V. The rise of hunger among low-income households: An analysis of the risks of food insecurity between 2004 and 2016 in a population-based study of UK adults. *J Epidemiol Community Health* 2019;**73**:668–73. doi:10.1136/jech-2018-211194

49 Tingay RS, Tan CJ, Tan NCW, *et al.* Food insecurity and low income in an English inner city. *J Public Health Med* 2003;**25**:156–9. doi:10.1093/pubmed/fdg032

50 Loopstra R. Vulnerability of Food Insecurity since COVID-19 Lockdown. 2020.

51 Koltai J, Toffolutti V, McKee M, *et al.* Changing probability of experiencing food insecurity by socioeconomic and demographic groups during the COVID-19 pandemic in the UK. *medRxiv* 2020;:0–18.

52 Pybus K, Power M, Pickett KE. 'We are constantly overdrawn, despite not spending money on anything other than bills and food': a mixed-methods, participatory study of food and food insecurity in the context of income inequality. *J Poverty Soc Justice* 2020;**xx**:1–22. doi:10.1332/175982720x15998354133521

53 MacLeod MA, Curl A, Kearns A. Understanding the Prevalence and Drivers of Food Bank Use: Evidence from Deprived Communities in Glasgow. *Soc Policy Soc* 2019;**18**:67–86. doi:10.1017/S1474746418000064

54 Bazerghi C, McKay FH, Dunn M. The Role of Food Banks in Addressing Food Insecurity: A Systematic Review. *J Community Health* 2016;**41**:732–40. doi:10.1007/s10900-015-0147-5

55 Caplan P. Big society or broken society? *Anthropol Today* 2016;**21**:443–55.http://www.journals.cambridge.org/abstract_S1474746415000184

56 POWER M, DOHERTY B, SMALL N, *et al.* All in it Together? Community Food Aid in a Multi-Ethnic Context. *J Soc Policy* 2017;**46**:447–71. doi:10.1017/S0047279417000010

57 Melchior M, Caspi A, Howard LM, *et al.* Mental health context of food insecurity: a representative cohort of families with young children. *Pediatrics* 2009;**124**:e564-72. doi:10.1542/peds.2009-0583

58 Harwood E. Discretion, dignity and choice : free school meals. 2018;:2018–20.

For peer review only - http://bmjopen.bmj.com/site/about/guidelines.xhtml

Acknowledgements

We would like to take this opportunity to thank The Food Foundation and ChildWise for allowing us to conduct analyses on their COVID-19 survey data and to the children and families who took part in the surveys.

Funding

This work is supported in part by the FixOurFood programme (BB/V004581/1) funded by the UK Research and Innovation (UKRI) Transforming Food Systems Programme; ActEarly UK Prevention Research Partnership Consortium (MR/S037527/1); The National Institute for Health Research under its Applied Research Collaboration Yorkshire and Humber (NIHR200166); and The Health Foundation COVID-19 Award (2301201).

Competing interests

All authors have completed the ICMJE uniform disclosure form at www.icmje.org/disclosure-of-interest/ and declare: support from the Bradford Institute for Health Research and the University of York; no financial relationships with any organisations that might have an interest in the submitted work in the previous three years; no other relationships or activities that could appear to have influenced the submitted work.

Transparency statement

The corresponding author (TY) affirms that the manuscript is an honest, accurate, and transparent account of the study being reported. No important aspects of the study have been omitted and any discrepancies from the study as originally planned have been disclosed.

Dissemination to participants and related patient and public communities

Findings will be disseminated through media outreach to the general public.

Contributors

TCY, MP, RHM, BL, WB, BD, and MB designed and planned the study. MB acquired the data. TCY performed the statistical analysis and was responsible for the initial draft of the manuscript. All authors were involved in interpreting the study results, revising the manuscript, and approving the final version for submission. MB is the guarantor. The corresponding author attests that all listed authors meet authorship criteria and that no others meeting the criteria have been omitted.

Ethics approval

We performed a secondary analysis of data that were collected externally by ChildWise (commissioned by the Food Foundation). We have discussed the approach taken by ChildWise extensively, who explained that ethical approval is only sought if they feel the questions or topic area are of a sensitive or personal nature. ChildWise surveys have “topic disclaimers” at the start and, where appropriate, respondents are provided with a list of helplines they could reach out to, and potentially sensitive questions include a “prefer not to say” option. When the research was commissioned it was not a pre-existing requirement from the client commissioning ChildWise. Informed consent was not obtained among child participants as parental participation in the survey and their agreement that a child within their household would complete the child’s portion of the survey was taken as assent. Data from this national survey are not identifiable.

For peer review only - http://bmjopen.bmj.com/site/about/guidelines.xhtml

Data availability statement
The datasets analysed during the current study are not freely available. Applications to access the data can be made to The Food Foundation.

The Corresponding Author has the right to grant on behalf of all authors and does grant on behalf of all authors, a worldwide licence to the Publishers and its licensees in perpetuity, in all forms, formats and media (whether known now or created in the future), to i) publish, reproduce, distribute, display and store the Contribution, ii) translate the Contribution into other languages, create adaptations, reprints, include within collections and create summaries, extracts and/or, abstracts of the Contribution, iii) create any other derivative work(s) based on the Contribution, iv) to exploit all subsidiary rights in the Contribution, v) the inclusion of electronic links from the Contribution to third party material where-ever it may be located; and, vi) licence any third party to do any or all of the above.

For peer review only

Table 1. Characteristics of the survey population.

	Total sample n=2166	
	N	Mean (SD)/%
**Parent responses**		
**Parent age**		
18-24	8	0.4
25-34	268	12.4
35-44	923	42.6
45-54	762	35.2
55-64	205	9.5
Missing	-	-
**Parent occupation**		
Higher	1341	61.9
Lower	825	38.1
Missing	-	-
**Geographical region**		
East Midlands	158	7.3
Eastern	196	9
London	282	13
North East	92	4.2
North West	240	11.1
Northern Ireland	73	3.4
Scotland	161	7.4
South East	300	13.9
South West	197	9.1
Wales	109	5
West Midlands	182	8.4
Yorkshire & Humberside	176	8.1
Missing	-	-
**Number in household**		
2	160	7.4
3	624	28.8
4	939	43.4
5	318	14.7
6+	125	5.8
Missing	-	-
**Child ethnicity**		
Asian	245	11.4
Other†	209	9.7
White	1691	78.8
Missing	21	-
**Child age (years)**	2166	12.4 (3.2)
Missing	-	-

For peer review only

**Child sex**		
Female	1076	49.7
Male	1090	50.3
Missing	-	-
**Child receives FSM**		
Yes	675	31.5
No	1467	68.5
Missing	24	-
**Child responses**		
**Potential food insecurity**		
Yes	431	20.6
No	1659	79.4
Missing	76	-
**Any food bank use**		
Yes	561	25.9
No	1605	74.1
Missing	-	-
**Food insecure***		
Yes	763	35.2
No	1403	64.8
Missing	-	-
**Find FSM embarrassing**		
Yes	62	9.7
No	578	90.3
Missing	1526	-
**Stressed/worried**ǂ		
Every/most days	236	18
Some/rarely	1053	82
Missing	19	-

†The Other ethnicity category includes the following groups: Black African, Black Caribbean, other Black background, mixed, and other background.

*Defined as responding affirmatively to any of the 6 potential food insecurity questions or indicated any food bank use

ǂResponses available only among a children participating in the January-February 2021 survey

For peer review only

Table 2. Food insecurity and food bank use by children who receive or do not receive free school meals.

	Receives FSM					Does not receive FSM				
	Food insecurity n=407 (60%)		No food insecurity n=268 (40%)			Food insecurity n=338 (23%)		No food insecurity n=1129 (77%)		
	N	Mean (SD)/%	N	Mean (SD)/%	*p*-value†	N	Mean (SD)/%	N	Mean (SD)/%	*p*-value†
**Parent age**					<0.001					<0.001
18-24	5	1.2	0	0		1	0.3	2	0.2	
25-34	91	22.4	44	16.4		43	12.7	87	7.7	
35-44	194	47.7	115	42.9		167	49.4	436	38.6	
45-54	97	23.8	75	28		107	31.7	474	41.9	
55-64	20	4.9	34	12.7		20	5.9	130	11.5	
**Parent occupation**					<0.001					0.1
Higher	238	58	106	40		214	63	754	68	
Lower	169	42	162	60		124	37	364	32	
**Number in household**					0.6					0.2
2	37	9.1	29	10.8		25	7.4	63	5.6	
3	123	30.2	80	29.9		106	31.4	311	27.5	
4	164	40.3	95	35.4		142	42	527	46.7	
5	51	12.5	43	16		46	13.6	177	15.7	
6+	32	7.9	21	7.8		19	5.6	51	4.5	
**Child ethnicity**					0.04					0.8
Asian	66	16	30	11		35	10.5	110	9.8	
Other	57	14	28	10		30	9	91	8.1	
White	282	70	210	78		268	80.5	916	82	
**Child age**	407	11.9 (3.1)	268	12.2 (3.2)	0.2	338	11.5 (3.1)	1129	12.9 (3.1)	<0.0001
**Child sex**					0.6					0.9

For peer review only - http://bmjopen.bmj.com/site/about/guidelines.xhtml

Female	182	45	126	47		176	52	580	51	
Male	225	55	142	53		162	48	549	49	
**Child finds FSM embarrassing***					<0.001					
No	313	86.9	233	95.5		-	-	-	-	
Yes	47	13.1	11	4.5		-	-	-	-	
**Child stressed/worried**					<0.001					<0.001
Every/most days	102	41	17	10		43	22	68	10	
Some/rarely	144	59	145	90		152	78	605	90	

†χ2, Fisher's exact, or Welch's two-sample t-test

*Only children responding affirmatively to receiving FSM were asked about this item

For peer review only

Figure 1. Percentage of children responding affirmatively to the six questions indicating potential food insecurity questions by FSM status.

Figure 2. Percentage of children responding affirmatively to two questions indicating food bank use by FSM status.

Figure 3. Probability of a child reporting feeling stressed or worried every day or most days in the past month by food security status.

Figure 4. Probability of a child reporting feeling stressed or worried every day or most days in the past month by food security and FSM status.

For peer review only

For peer review only - http://bmjopen.bmj.com/site/about/guidelines.xhtml

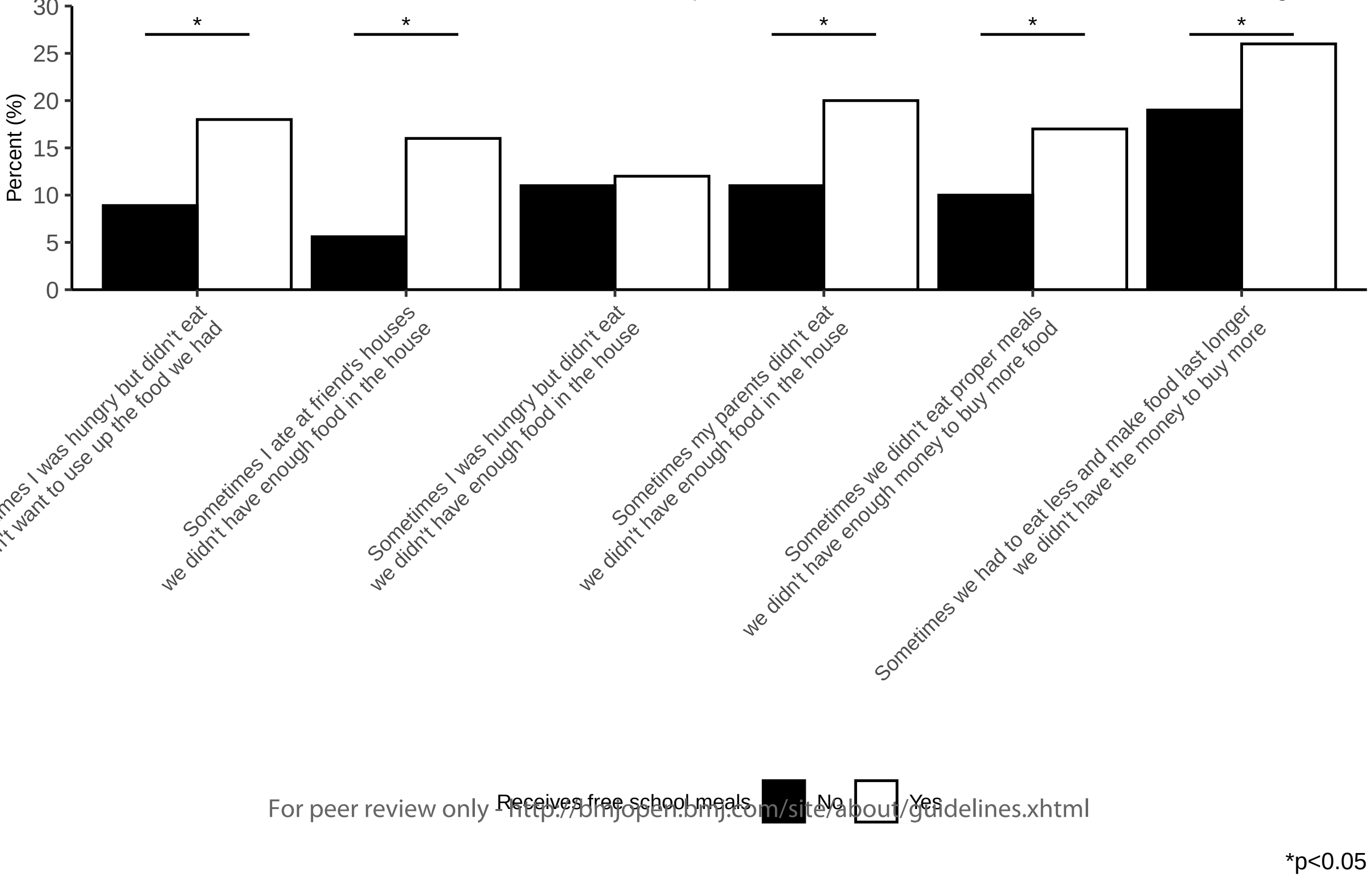
Percent (%)
30
25
20
15
10
5
0
*
*
*
*
*
Sometimes I was hungry but didn't eat
I didn't want to use up the food we had
Sometimes I ate at friend's houses
we didn't have enough food in the house
Sometimes I was hungry but didn't eat
we didn't have enough food in the house
Sometimes my parents didn't eat
we didn't have enough food in the house
Sometimes we didn't eat proper meals
we didn't have enough money to buy more food
Sometimes we had to eat less and make food last longer
we didn't have the money to buy more
Receives free school meals
No
Yes
*p<0.05

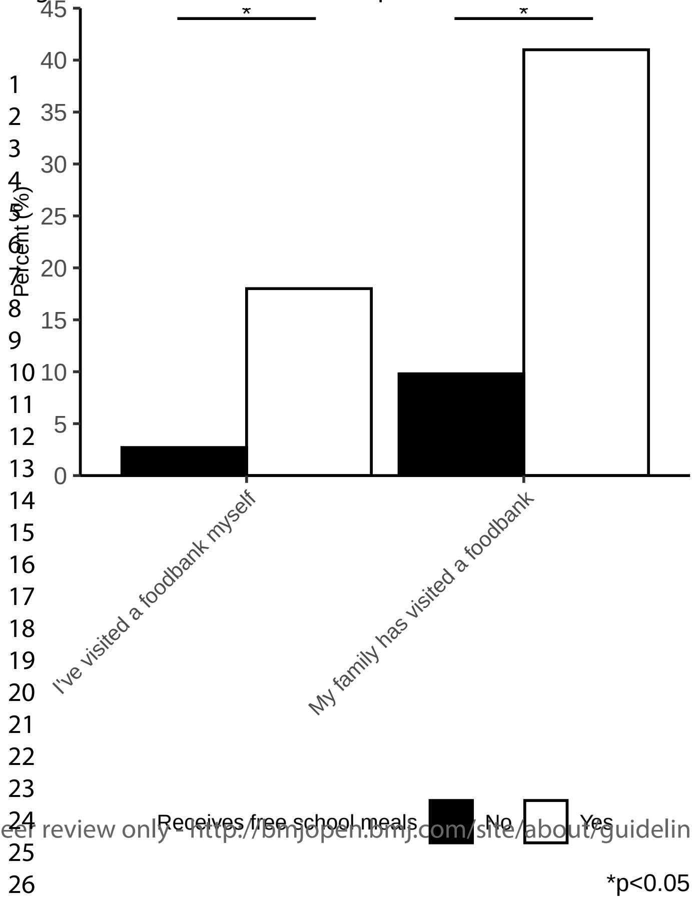

peer review only - http://bmjopen.bmj.com/site/about/guidelines.xh

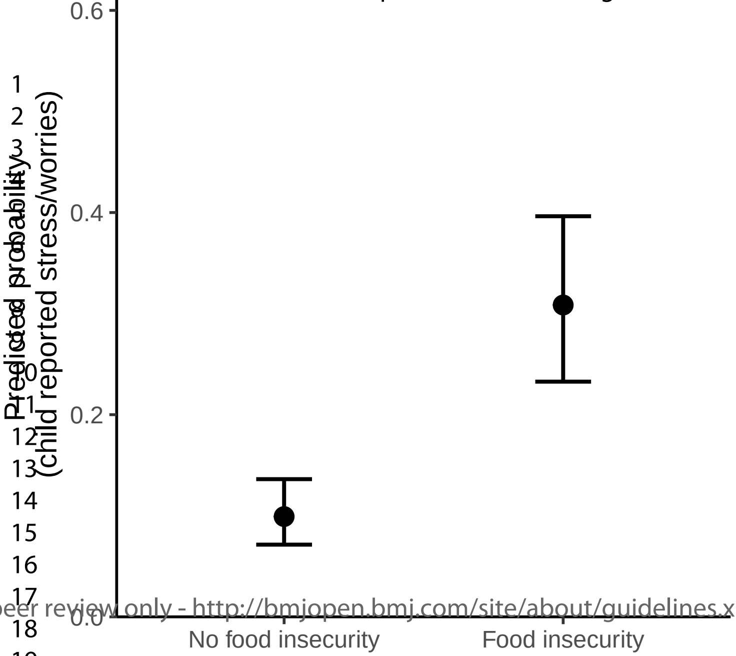

peer review only - http://bmjopen.bmj.com/site/about/guidelines.xh

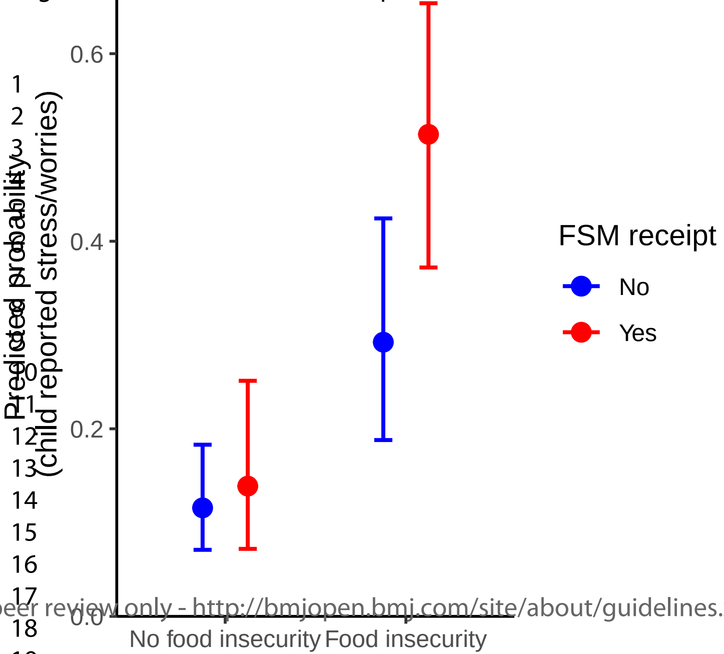

peer review only - http://bmjopen.bmj.com/site/about/guidelines.xh

Supplemental file 1. Characteristics of the survey population by participants responding to both surveys or to the first survey period (August-September 2020).

	Total sample n=2166		August-September 2020 only N=858		Responded to both surveys N=206		
	N	Mean (SD)/%	N	Mean (SD)/%	N	Mean (SD)/%	p-value
**Parent responses**							
**Parent age**							**0.07**
18-24	8	0.4	1	0.1	0	0	
25-34	268	12.4	112	13.1	18	8.7	
35-44	923	42.6	377	43.9	78	37.9	
45-54	762	35.2	292	34	88	42.7	
55-64	205	9.5	76	8.9	22	10.7	
Missing	-	-	-	-	-	-	
**Parent occupation**							**0.4**
Higher	1341	61.9	556	65	126	61	
Lower	825	38.1	302	35	80	39	
Missing	-	-	-	-	-	-	
**Geographical region**							**0.05**
East Midlands	158	7.3	68	7.9	6	2.9	
Eastern	196	9	72	8.4	20	9.7	
London	282	13	117	13.6	20	9.7	
North East	92	4.2	38	4.4	7	3.4	
North West	240	11.1	95	11.1	25	12.1	
Northern Ireland	73	3.4	27	3.1	9	4.1	
Scotland	161	7.4	59	6.9	25	12.1	
South East	300	13.9	122	14.2	26	12.6	
South West	197	9.1	76	8.9	15	7.3	
Wales	109	5	43	5.0	13	6.3	
West Midlands	182	8.4	57	7.8	24	11.7	
Yorkshire & Humberside	176	8.1	74	8.6	16	7.8	
Missing	-	-	-	-	-	-	
**Number in household**							**0.4**
2	160	7.4	66	7.7	8	3.9	
3	624	28.8	248	28.9	62	30.1	
4	939	43.4	361	42.1	89	43.2	
5	318	14.7	124	14.5	33	16	
6+	125	5.8	59	6.9	14	6.8	
Missing	-	-	-	-	-	-	
**Child ethnicity**							**0.6**
Asian	245	11.4	95	11.1	22	10.8	
Other†	209	9.7	69	8.1	21	10.3	
White	1691	78.8	687	80.8	161	78.9	
Missing	21	-	8	-	2	-	

For peer review only

**Child age (years)**	2166	12.4 (3.2)	858	12.4 (3.2)	206	12.5 (3.2)	**0.7**
Missing	-	-	-	-	-	-	
**Child sex**							**0.4**
Female	1076	49.7	422	49	109	53	
Male	1090	50.3	436	51	97	47	
Missing	-	-	-	-	-	-	
**Child receives FSM**							**0.08**
Yes	675	31.5	260	31	50	24	
No	1467	68.5	587	79	156	76	
Missing	24	-	11	-	-	-	
**Child responses**							
**Potential food insecurity**							**0.2**
Yes	431	20.6	165	20	31	15	
No	1659	79.4	667	80	171	85	
Missing	76	-	26	-	4	-	
**Any food bank use**							**0.002**
Yes	561	25.9	224	26	32	16	
No	1605	74.1	634	74	174	84	
Missing	-	-	-	-	-	-	
**Food insecure***							**0.01**
Yes	763	35.2	298	35	52	25	
No	1403	64.8	560	65	154	75	
Missing	-	-	-	-			
**Find FSM embarrassing**							**0.8**
Yes	62	9.7	26	11	4	8.2	
No	578	90.3	214	89	45	91.8	
Missing	1526	-	618	-	157	-	

†The Other ethnicity category includes the following groups: Black African, Black Caribbean, other Black background, mixed, and other background.

*Defined as responding affirmatively to any of the 6 potential food insecurity questions or indicated any food bank use

For peer review only

Supplemental file 2. Characteristics of the survey population by survey period

	Total sample n=2166		August-September 2020 N=858		January-February 2021 N=1308		
	N	Mean (SD)/%	N	Mean (SD)/%	N	Mean (SD)/%	p-value
**Parent responses**							
**Parent age**							**0.3**
18-24	8	0.4	1	0.1	7	0.5	
25-34	268	12.4	112	13.1	156	11.9	
35-44	923	42.6	377	43.9	546	41.7	
45-54	762	35.2	292	34	470	35.9	
55-64	205	9.5	76	8.9	129	9.9	
Missing	-	-	-	-	-	-	
**Parent occupation**							**0.03**
Higher	1341	61.9	556	65	785	60	
Lower	825	38.1	302	35	423	40	
Missing	-	-	-	-	-	-	
**Geographical region**							**1**
East Midlands	158	7.3	68	7.9	90	6.9	
Eastern	196	9	72	8.4	124	9.5	
London	282	13	117	13.6	165	12.6	
North East	92	4.2	38	4.4	54	4.1	
North West	240	11.1	95	11.1	145	11.1	
Northern Ireland	73	3.4	27	3.1	46	3.5	
Scotland	161	7.4	59	6.9	102	7.8	
South East	300	13.9	122	14.2	178	13.6	
South West	197	9.1	76	8.9	121	9.3	
Wales	109	5	43	5.0	66	5.0	
West Midlands	182	8.4	57	7.8	115	8.8	
Yorkshire & Humberside	176	8.1	74	8.6	102	7.8	
Missing	-	-	-	-	-	-	
**Number in household**							**0.4**
2	160	7.4	66	7.7	94	7.2	
3	624	28.8	248	28.9	376	28.7	
4	939	43.4	361	42.1	578	44.2	
5	318	14.7	124	14.5	194	14.8	
6+	125	5.8	59	6.9	66	5.0	
Missing	-	-	-	-	-	-	
**Child ethnicity**							**0.1**
Asian	245	11.4	95	11.1	151	12	
Other†	209	9.7	69	8.1	140	11	
White	1691	78.8	687	80.8	1004	78	
Missing	21	-	8	-	13	-	
**Child age (years)**	2166	12.4 (3.2)	858	12.4 (3.2)	1308	12.4 (3.2)	

For peer review only

Missing	-	-	-	-	-	-	
**Child sex**							**0.7**
Female	1076	49.7	422	49	654	50	
Male	1090	50.3	436	51	654	50	
Missing	-	-	-	-	-	-	
**Child receives FSM**							**0.5**
Yes	675	31.5	260	31	415	32	
No	1467	68.5	587	79	880	68	
Missing	24	-	11	-	13	-	
**Child responses**							
**Potential food insecurity**							**0.5**
Yes	431	20.6	165	20	266	21	
No	1659	79.4	667	80	992	79	
Missing	76	-	26	-	60	-	
**Any food bank use**							**0.9**
Yes	561	25.9	224	26	337	26	
No	1605	74.1	634	74	971	74	
Missing	-	-	-	-	-	-	
**Food insecure***							**0.7**
Yes	763	35.2	298	35	465	36	
No	1403	64.8	560	65	843	64	
Missing	-	-	-	-	-	-	
**Find FSM embarrassing**							**0.3**
Yes	62	9.7	26	11	36	9.0	
No	578	90.3	214	89	364	91	
Missing	1526	-	618	-	908	-	
**Stressed/worried**ǂ							-
Every/most days	236	18	-	-	236	18	
Some/rarely	1053	82	-	-	1053	82	
Missing	19	-	-	-	19	-	

†The Other ethnicity category includes the following groups: Black African, Black Caribbean, other Black background, mixed, and other background.

*Defined as responding affirmatively to any of the 6 potential food insecurity questions or indicated any food bank use

ǂResponses available only among a children participating in the January-February 2021 survey.

p-value differences between survey periods

For peer review only

Supplementary file 3. DAG for food insecurity and child mental health.

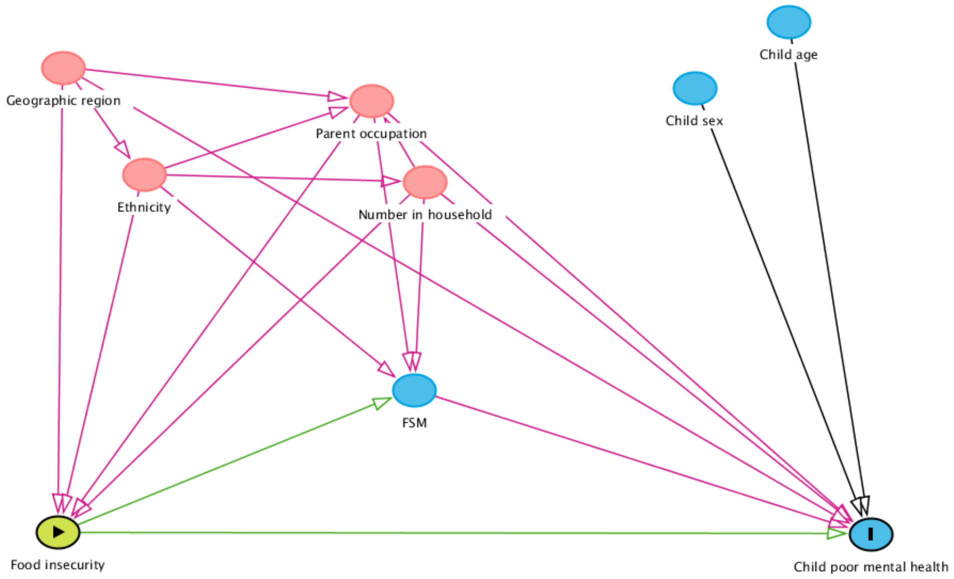

STROBE Statement—Checklist of items that should be included in reports of ***cross-sectional studies***

	Item No	Recommendation
**Title and abstract**	1	(*a*) Indicate the study's design with a commonly used term in the title or the abstract page 1
		(*b*) Provide in the abstract an informative and balanced summary of what was done and what was found page 1-2
**Introduction**		
Background/rationale	2	Explain the scientific background and rationale for the investigation being reported page 3-4
Objectives	3	State specific objectives, including any prespecified hypotheses page 4
**Methods**		
Study design	4	Present key elements of study design early in the paper page 4
Setting	5	Describe the setting, locations, and relevant dates, including periods of recruitment, exposure, follow-up, and data collection page 4
Participants	6	(*a*) Give the eligibility criteria, and the sources and methods of selection of participants page 4
Variables	7	Clearly define all outcomes, exposures, predictors, potential confounders, and effect modifiers. Give diagnostic criteria, if applicable pages 4-6
Data sources/ measurement	8*	For each variable of interest, give sources of data and details of methods of assessment (measurement). Describe comparability of assessment methods if there is more than one group pages 4-6
Bias	9	Describe any efforts to address potential sources of bias page 6
Study size	10	Explain how the study size was arrived at page 6
Quantitative variables	11	Explain how quantitative variables were handled in the analyses. If applicable, describe which groupings were chosen and why pages 4-6
Statistical methods	12	(*a*) Describe all statistical methods, including those used to control for confounding pages 5-6
		(*b*) Describe any methods used to examine subgroups and interactions pages 6
		(*c*) Explain how missing data were addressed page 6
		(*d*) If applicable, describe analytical methods taking account of sampling strategy pages 4,6
		(*e*) Describe any sensitivity analyses
**Results**		
Participants	13*	(a) Report numbers of individuals at each stage of study—eg numbers potentially eligible, examined for eligibility, confirmed eligible, included in the study, completing follow-up, and analysed page 4,6
		(b) Give reasons for non-participation at each stage page 4
		(c) Consider use of a flow diagram
Descriptive data	14*	(a) Give characteristics of study participants (eg demographic, clinical, social) and information on exposures and potential confounders page 7
		(b) Indicate number of participants with missing data for each variable of interest page 16-17
Outcome data	15*	Report numbers of outcome events or summary measures page 6-7
Main results	16	(*a*) Give unadjusted estimates and, if applicable, confounder-adjusted estimates and their precision (eg, 95% confidence interval). Make clear which confounders were adjusted for and why they were included pages 6-7

For peer review only

		(*b*) Report category boundaries when continuous variables were categorized
		(*c*) If relevant, consider translating estimates of relative risk into absolute risk for a meaningful time period
Other analyses	17	Report other analyses done—eg analyses of subgroups and interactions, and sensitivity analyses page 7
**Discussion**		
Key results	18	Summarise key results with reference to study objectives pages 6-7
Limitations	19	Discuss limitations of the study, taking into account sources of potential bias or imprecision. Discuss both direction and magnitude of any potential bias pages 9-10
Interpretation	20	Give a cautious overall interpretation of results considering objectives, limitations, multiplicity of analyses, results from similar studies, and other relevant evidence pages 7-10
Generalisability	21	Discuss the generalisability (external validity) of the study results page 8-10
**Other information**		
Funding	22	Give the source of funding and the role of the funders for the present study and, if applicable, for the original study on which the present article is based page 14

*Give information separately for exposed and unexposed groups.

Note: An Explanation and Elaboration article discusses each checklist item and gives methodological background and published examples of transparent reporting. The STROBE checklist is best used in conjunction with this article (freely available on the Web sites of PLoS Medicine at http://www.plosmedicine.org/, Annals of Internal Medicine at http://www.annals.org/, and Epidemiology at http://www.epidem.com/). Information on the STROBE Initiative is available at www.strobe-statement.org.

For peer review only

For peer review only - http://bmjopen.bmj.com/site/about/guidelines.xhtml
